# Contaminated Tijuana River Contributes to Regional Particulate Matter (PM) Levels Highlighting Overlooked Water–Air Transfer of Gaseous PM Precursors

**DOI:** 10.1021/acs.est.5c08369

**Published:** 2025-12-16

**Authors:** Karolina Cysneiros de Carvalho, Kelley C. Barsanti, Justin D. Hamlin, Kimberly A. Prather, William C. Porter

**Affiliations:** † Department of Environmental Sciences, 415778 University of California Riverside, Riverside, California 92521, United States; ‡ Atmospheric Chemistry Observations & Modeling Laboratory, NSF National Center for Atmospheric Research, Boulder, Colorado 80305, United States; § Department of Chemistry and Biochemistry, 8784 University of California San Diego, La Jolla, California 92093, United States; ∥ Scripps Institution of Oceanography, 8784 University of California San Diego, La Jolla, California 92037, United States

**Keywords:** PM, VOCs, secondary aerosol, low-cost sensors, air quality, water pollution, water−air partitioning, urban-coastal interface

## Abstract

The San Diego–Tijuana border region has experienced rapid urbanization and industrial growth with unmitigated environmental consequences. For nearly a century, the Tijuana River (TR) has carried untreated sewage and industrial waste into the United States, contributing to a long-recognized and ongoing water pollution crisis. However, the impact of this pollution on air quality has been almost entirely overlooked until very recently. Analysis of low-cost air sensor data reveals that gases released from the polluted TR directly contribute to the formation of fine aerosols, increasing PM_1_ (particulate matter <1 μm) concentrations, particularly when river flow is high and atmospheric dispersion is low. Analysis of PM_1_ size distributions revealed the enhancement of smaller particle fractions, and persistently high PM_1_-to-PM_2.5_ ratios (≥0.56 ± 0.15) showed that submicrometer particles constitute the majority of fine PM mass. Combined with recent evidence of elevated gas-phase emissions from the polluted TR, these results point to secondary aerosol formation—driven by the chemical transformations of river-emitted gaseous precursors—as a major source of PM_1._ Concentrations peaked near a turbulent riverine hotspot, particularly at night when intensified flow and stable conditions promoted secondary aerosol formation. These findings identify a previously unrecognized source of urban air pollution, showing that the river-to-air transfer of particulate precursors can perpetuate poor air quality and heighten environmental justice and public health concerns.

## Graphical abstract

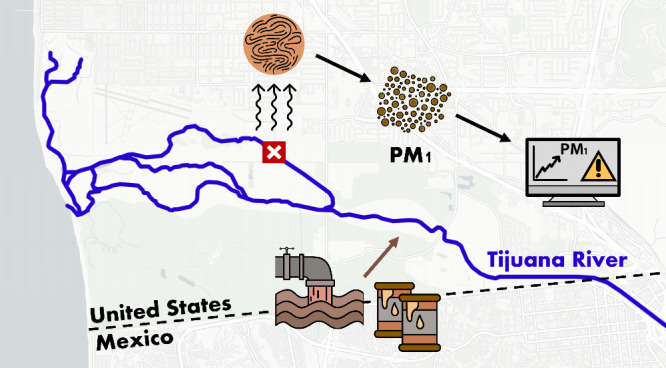

## 1 Introduction

The Tijuana River (TR) flows through a binational watershed, with about 75% of its drainage area located in Mexico and the remaining 25% within the United States (Figure 1B).^1^ The rapid industrial growth of the Mexican city of Tijuana,^2^ combined with poor wastewater management, has significantly affected the TR, leading to a continuous influx of urban runoff, industrial effluents, and untreated sewage into the river.^2–4^ These contaminated waters traverse the border and ultimately discharge into the Pacific Ocean, just south of Imperial Beach (IB), in the State of California. Focusing on the TR, this study examines how urban particulate matter (PM) levels are influenced by the chemical transformation of gaseous emissions from polluted waterways, leading to secondary aerosol (SA) formation.

**Figure 1:**
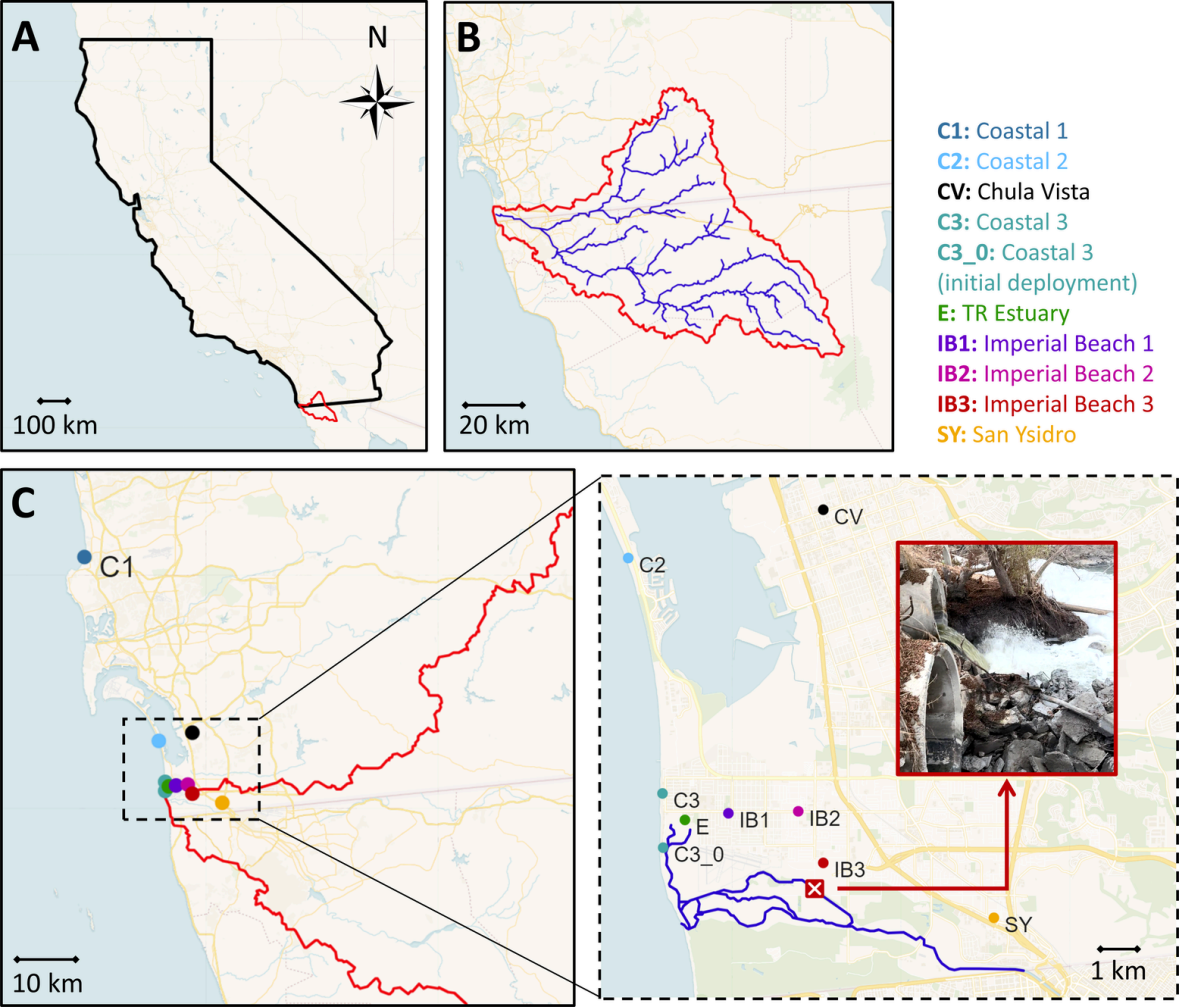
Map of the study domain. Panel (A) delineates the state of California in black and the Tijuana River (TR) watershed in red. Panel (B) shows a detailed view of the drainage area of the watershed. Panel (C) displays the specific locations of the low-cost sensors deployed as part of the air quality monitoring network established for this study. The Panel (C) inset shows the TR path in blue and the location of the turbulent hotspot indicated by a red ×-marker and the inset photograph.

In 1993, the California State Water Control Board reported that IB experienced 187 days of beach closures due to sewage contamination from the TR.^5^ In 1997, the South Bay International Wastewater Treatment Plant (SBIWTP) was constructed at the border to divert and treat some of Tijuana’s sewage.^6^ This facility has consistently failed to meet Clean Water Act (CWA) standards^4,6^ and frequently operates beyond its designed capacity of 95 million liters per day.^7^ Thus, for many years following its establishment, potentially hazardous waste has infiltrated the TR.^8,9^ In 2017, damage to Tijuana’s sewage system spilled an estimated 541 million liters of untreated wastewater into the river,^10^ closing beaches from Rosarito, Baja California, to Coronado, California.^11^ Due to the continued wastewater discharges, the southernmost stretch of IB’s coastline (the Tijuana Slough shoreline) has remained closed since December 2021,^12^ underscoring the region’s enduring transboundary pollution crisis.

Many studies have highlighted how the ongoing contamination of the TR has compromised the soil and water quality in South San Diego County. A myriad of pathogens has been reported in the TR and coastal waters of South San Diego County.^8,13–15^ Persistent, bioaccumulative, and toxic (PBT) chemicals, including organochlorine pesticides banned in the United States, have been found in sediment samples collected within the boundaries of the TR Estuary.^16^ Furthermore, concentrations of SARS-CoV-2 RNA comparable to those found in untreated wastewater were detected in transboundary flows upstream of the Mexico-United States border.^17^

The literature on the impact of polluted water on air quality in South San Diego County, however, is sparse. The Cross Surfzone/Inner-shelf Dye Exchange (CSIDE) experiment conducted in IB was the first study to positively demonstrate that contaminated coastal waters have the potential to expose inland residents to airborne water pollution.^18^ A follow-up study confirmed, using genomic sequencing, that sewage-related bacteria in the TR are transferred to land in sea spray aerosol.^19^ More recently, high wastewater-polluted TR flows were associated with sustained concentrations of hydrogen sulfide (H_2_S) in the County that exceeded ambient standards set by the California Air Resources Board, and emissions of many volatile organic compounds (VOCs) with known toxicity.^20^

Communities living in South San Diego County have reported adverse health effects associated with this transboundary pollution crisis^20,21^ which may arise not only from direct exposure to the wastewater but also from inhalation exposure to pollutants emitted and formed from these contaminated waters. Volatile compounds can serve as precursors to criteria pollutants, such as ozone and PM. Increases in PM levels specifically occur when atmospheric chemical reactions of gaseous compounds form lower-volatility compounds that are able to partition to the particle phase,^22^ thereby producing SA and contributing to PM. PM is regulated by the United States Environmental Protection Agency (EPA)^23^ and affects life quality and expectancy by enhancing the risk of cancer and cardiopulmonary diseases.^24,25^

A survey of published literature suggests that no studies have been performed that explore the influence of gaseous emissions from contaminated water bodies on urban PM concentrations. VOC fluxes from contaminated rivers in China have been measured; however, there was no assessment of their correlation with PM levels.^26^ Secondary organic aerosol (SOA) formation potential from VOC emissions associated with waste containment ponds was assessed by Drollette et al.,^27^ but no connection with urban PM levels was established. This study investigated the spatial-temporal variability of PM_1_ (i.e., particles with an aerodynamic diameter less than 1.0 μm) across South San Diego County (Figure 1C). Combining measurements from low-cost air quality sensors with local meteorological data, this work provides evidence that the TR significantly contributes to increases in PM_1_ levels in the region, primarily driven by the chemical transformation of gaseous compounds originating from the polluted river.

## 2 Materials and Methods

### 2.1 Site Description

Air quality sensors were deployed at 22 locations in South San Diego County beginning in May 2024. Rico et al.^20^ have recently demonstrated how H_2_S emissions in the region originate from a turbulent and foamy site along the TR. Expanding upon that finding, this study investigates the role of the TR riverine hotspot as a key point source contributing to the observed PM levels. Nine monitoring locations were strategically chosen to explore the effects of polluted transboundary flows on coastal and inland particulate pollution while also considering the influence of meteorology on pollutant concentrations. Each monitoring location (hereinafter termed a site) is described below, and measurement details are discussed in the following section.

The locations of the sites are shown in Figure 1 with respect to the TR watershed domain and the riverine hotspot, which includes three coastal sites (labeled C1, C2, and C3) and six inland sites (labeled SY, CV, E, IB1, IB2, and IB3). The northernmost site, C1, was located at the Scripps Institution of Oceanography pier in La Jolla, San Diego. Coastal site C2 was located at Silver Strand Beach in Coronado, approximately 30 km south of C1. Site C3, located in IB, was the closest coastal site to the TR and its outfall, though still 4 km northwest of the riverine hotspot. The air quality sensor at site C3 was initially deployed at the top of a four-story building (indicated as site C3_0 in Figure 1), 1.2 km from its final location. Data from this initial deployment period are included in this study, and it will be explicitly mentioned when each data set is discussed.

The San Ysidro site (SY) was located approximately 1.5 km northwest of where the TR crosses the international border. This site was surrounded by busy road arteries, including Interstate 5 (I-5) to the west, Interstate 805 (I-805) to the east, and State Route 905 (SR-905) to the north. All three highways were within 2 km of SY, subjecting this site to significant traffic emissions. The Chula Vista site (CV) was located in a residential area approximately 3 km east of San Diego Bay and adjacent to I-5, I-805, and State Route 54 (SR-54). Therefore, a mixed oceanic-urban pollution profile would be expected at this location.

In the IB region, one site was located at the northernmost part of the Tijuana Slough National Wildlife Refuge, facing the wetlands (site E). Thus, riverine and surrounding vegetation emissions were likely dominant sources at this location. The sites IB1, IB2, and IB3 represent urban areas in the region. Sites IB1 and IB2 were located along Imperial Beach Boulevard, while site IB3 was located in a residential neighborhood closest to the riverine hotspot. Thus, sites C1, C2, CV, and SY are the farthest from the TR hotspot, while sites C3, E, IB1, IB2, and IB3 are progressively closer. Table S1 provides a summary of the sites, including their geographic coordinates, deployment dates, and data acquired.

### 2.2 Instrumentation and Measurements

#### 2.2.1 Atmospheric Particle Matter (PM)

PM data were acquired using two models of low-cost sensors made by QuantAQ Inc. (Somerville, MA, USA). The MODULAIR-PM provided minute-resolution measurements of PM_1_, PM_2.5_, and PM_10_ (i.e., mass concentrations of particles with an aerodynamic diameter less than 1.0, 2.5, and 10 μm, respectively) along with particle size distributions from 0.35 to 40.0 μm subdivided into 24 bins. The MODULAIR allowed the additional monitoring of ozone (O_3_), nitrogen oxide (NO), nitrogen dioxide (NO_2_), carbon monoxide (CO), and carbon dioxide (CO_2_) mixing ratios; however, only the PM_1_ and PM_2.5_ data sets are considered in this work.

Both sensors employ the same technology for particle measurements, which integrates nephelometry with single particle scattering, enabling the measurement of aerosol loadings across different size ranges.^28,29^ Relative humidity (RH) is a significant source of uncertainty for optical particle sensors, particularly when measuring hygroscopic aerosols in RH conditions above the deliquescence point.^30^ Consequently, the EPA requires PM_2.5_ measurements to be conducted under dry conditions, specifically with RH levels below 40%.^31,32^ While the low-cost sensors deployed do not sample dried ambient air, they include a manufacturer-applied correction to account for hygroscopic growth, and previous studies have demonstrated the high accuracy of MODULAIR monitors in reporting PM mass concentrations across a range of RH values.^33–35^

The performance of the QuantAQ sensors under the study conditions was evaluated by comparing data from the QuantAQ installed at the inland monitoring site (IB3) with collocated measurements of dried aerosols (RH < 45%) using a research-grade Optical Particle Sizer (OPS, Model 3330, TSI, Inc., Shoreview, MN). The analysis considered only the MODULAIR Optical Particle Counter (OPC), excluding nephelometer estimates, enabling a comparison that aligns with the OPS sizing measurements. OPS number size distributions were used to estimate PM_1_ following the method outlined by Hagan and Cross.^29^ Since the OPS measured dried aerosols, no correction for hygroscopic growth was applied. The results illustrated in Figure S1 show a correlation coefficient between the low-cost OPC and the high-grade OPS for PM_1_ mass concentration estimations equal to 0.59, with a slope of 0.91. Removing measurements obtained at RH > 70% increased the regression coefficient to 0.83 with a slope of 0.76, and illustrates the influence of RH on the PM_1_ estimates. This satisfactory agreement supports the use of QuantAQ data in this study.

#### 2.2.2 Meteorological Parameters and TR Transboundary Flows

Meteorology strongly influences the distribution of atmospheric pollutants.^36^ Extensive research has demonstrated relationships between PM concentrations and various meteorological parameters, including temperature, RH, wind speed, wind direction, mixing layer height (MLH), precipitation, cloud cover, and solar irradiance.^37–42^ Here, real-time measurements of ambient temperature and RH were provided by the QuantAQs at a precision of ±0.2 °C and ±2%, respectively.^28^ Sonic anemometers (Vantage Pro2TM, Davis Instruments, Hayward, CA, USA) were mounted on top of all MODULAIR monitors, allowing for localized air displacement measurements.

Atmospheric vertical temperature gradients were used as a proxy to understand the extent of vertical mixing during different periods of the study. Temperature inversion layers are defined as an atmospheric layer wherein temperature increases with height.^43,44^ This phenomenon is characterized by increased atmospheric stability and lower MLHs, which hinder vertical mixing and trap pollutants near the surface.^38,41,44^ To examine the role of atmospheric stability on air quality, the temperature difference between 800 and 40 m (d*T*) was used to identify the presence of inversions within the planetary boundary layer. Regional temperature vertical profiles needed for d*T* calculations were obtained from the National Oceanic and Atmospheric Administration/Earth System Research Laboratories Physical Sciences Laboratory Web-based Reanalysis Intercomparison Tool (WRIT) using the NCEP-NCAR Reanalysis 1 data set.^45^

The effect of the river on PM levels was investigated by considering transboundary flow measurements retrieved from the United States Section of the International Boundary and Water Commission (USIBWC) database,^46^ the federal agency responsible for implementing boundary and water treaties between the United States and Mexico. A flow gauge positioned in the river approximately 1 km east of the SBIWTP and 0.2 km from the international border granted the TR discharge rates used in this analysis.

### 2.3 Data Analysis

Data collected from September 1st to November 30th, 2024, were used in this study. The time series of daily d*T* estimations and TR transboundary flows is illustrated in Figure 2. d*T* estimations were calculated at 05:00 local time (LT). On the heatmap, elevated TR discharge rates and higher flows are represented by warmer colors, while cooler colors represent lower flows. Diurnal variations of transboundary flows can be further analyzed by examining the color shift along the vertical axis. Due to the high flow variability observed during the study, the color scale is maximized at a discharge rate of 3.0 m^3^ s^–1^, facilitating the identification of high and low flow periods. However, the maximum transboundary flow rate recorded during this period was equal to 5.3 m^3^ s^–1^ at a 15 min time resolution, as displayed in Cartesian coordinates in Figure S2.

**Figure 2:**
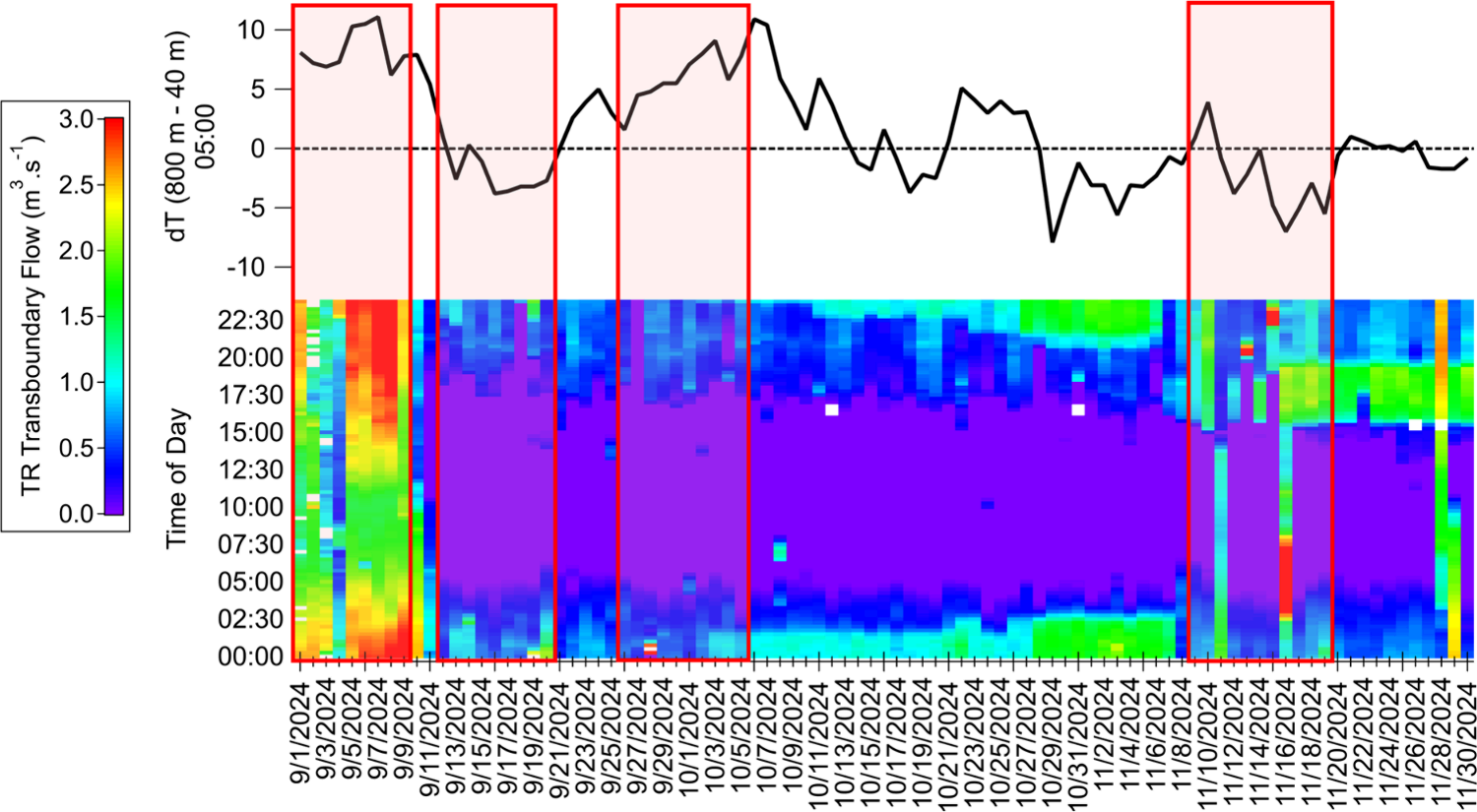
Daily estimates of d*T* (top) and 15 min resolved heat map illustrating time series of Tijuana River (TR) transboundary flow rates (bottom) from September 1st to November 30th, 2024. d*T* denotes the difference in vertical temperature between 800 and 40 m at 05:00 LT. Red boxes delineate the time periods defined to investigate the influence of TR transboundary flows on PM_1_ levels.

High discharge rates were consistently observed throughout the first week of September until the Mexican pump station PBCILA returned to operation on September 10th.^47^ During this period, d*T* was positive and indicative of limited vertical dispersion. The subsequent period of decreased flow coincided with improved mixing, as indicated by significantly lower d*T* values. During this time, near-zero flows were recorded during the day. Throughout September and October, regardless of the difference in flow rates, a diurnal pattern was evident with the highest flows at night. By mid-November, discharge rates were distinctly more variable, with some days experiencing nonzero continuous flow and peaks occurring in the early evenings.

The data were separated into four different periods of 9 to 11 days that represented distinct combinations of TR discharge rates and atmospheric mixing conditions, as outlined in Table 1. Period 1 was characterized by sustained high flow rates, low MLHs, and elevated ambient temperatures. Period 2 exhibited a decrease in discharge rates, accompanied by an increase in MLHs and lower temperatures compared to Period 1. During Period 3, temperatures and transboundary flows were comparable to Period 2, but vertical mixing was limited due to lower MLHs. Period 4 was marked by low temperatures and increased, yet highly variable, discharge rates and MLHs, as depicted in the boxplot shown in Figure S3. Diurnal profiles of recorded ambient temperatures, RH, and transboundary flow rates during each period are displayed in Figures S4–S6. Additionally, wind observations at each available site are plotted for each period in Figures S7–S14.

**Table 1:** Summary of the Different Time Periods Defined To Investigate the Influence of the Tijuana River Transboundary Flows on PM_1_ Levels^a^

	start period	end period	temp. (°C)	RH (%)	d*T* (m)	TR flow(m^3^ s^–1^)	description
**1**	9/1/24	9/9/24	26.1 (20.3–35.5)	69.7 (45.6–84.6)	8.4 (6.2–11.1)	1.9 (0.3–3.4)	low MLH high flow
**2**	9/12/24	9/20/24	22.9 (17.5–28.8)	60.8 (39.6–75.7)	–2.1 (−3.8 to 1)	0.2 (0–2.2)	high MLH low flow
**3**	9/26/24	10/5/24	21.2 (17.3–26.7)	73.6 (54.7–85)	6.0 (1.6–9.1)	0.2 (0–2.9)	low MLH low flow
**4**	11/9/24	11/19/24	17.0 (8.5–27.8)	58.2 (20.2–81.3)	–2.5 (−7 to 3.9)	0.6 (0–5.3)	high MLH high flow

a Notes: Temp., RH, d*T*, and TR Flow values averaged between the specified start and end dates. Values in parentheses represent maximum and minimum observations. Temp. and RH values were averaged across all monitoring stations prior to the time-averaging calculations. Abbreviations: Temp., temperature; RH, relative humidity; TR, Tijuana River.

To ensure that the vertical temperature difference, d*T*, is a valid proxy to represent MLHs, reanalysis planetary boundary layer heights (PBLHs) from the ERA5 database^48^ were compared against d*T* calculations. ERA5 defines PBLH as “the depth of air next to the Earth’s surface which is most affected by the resistance to the transfer of momentum, heat or moisture across the surface” and their data align with MLH ceilometer measurements.^49^ The comparison (shown in Figures S15–S18) demonstrates the suitability of using d*T* estimations to identify different vertical dispersion conditions in the atmosphere. Ground-level temperature and wind diurnal profiles retrieved from the ERA5 database are also plotted for each defined period.

## 3 Results and Discussion

### 3.1 PM_1_ Spatial and Temporal Variability

Boxplots summarizing daytime (06:00–19:59) and nighttime (20:00–05:59) PM_1_ observations across all sites for each defined period are presented in Figure 3. Horizontal lines represent median concentrations, with notches symbolizing the 95% confidence interval. Lower and upper whiskers denote the 9th and 91st percentiles, respectively. The sites are systematically organized on the *x*-axis by placing the most northern sites at the beginning, followed by the southern sites. This arrangement displays the sites located in the IB region (i.e., sites C3, E, IB1, IB2, and IB3) in order of decreasing distance from the turbulent TR hotspot, which is indicated by the red “-×” in Figure 1. These PM_1_ distributions emphasize the substantial spatial-temporal variability observed during the selected weeks within the three-month sampling period.

**Figure 3:**
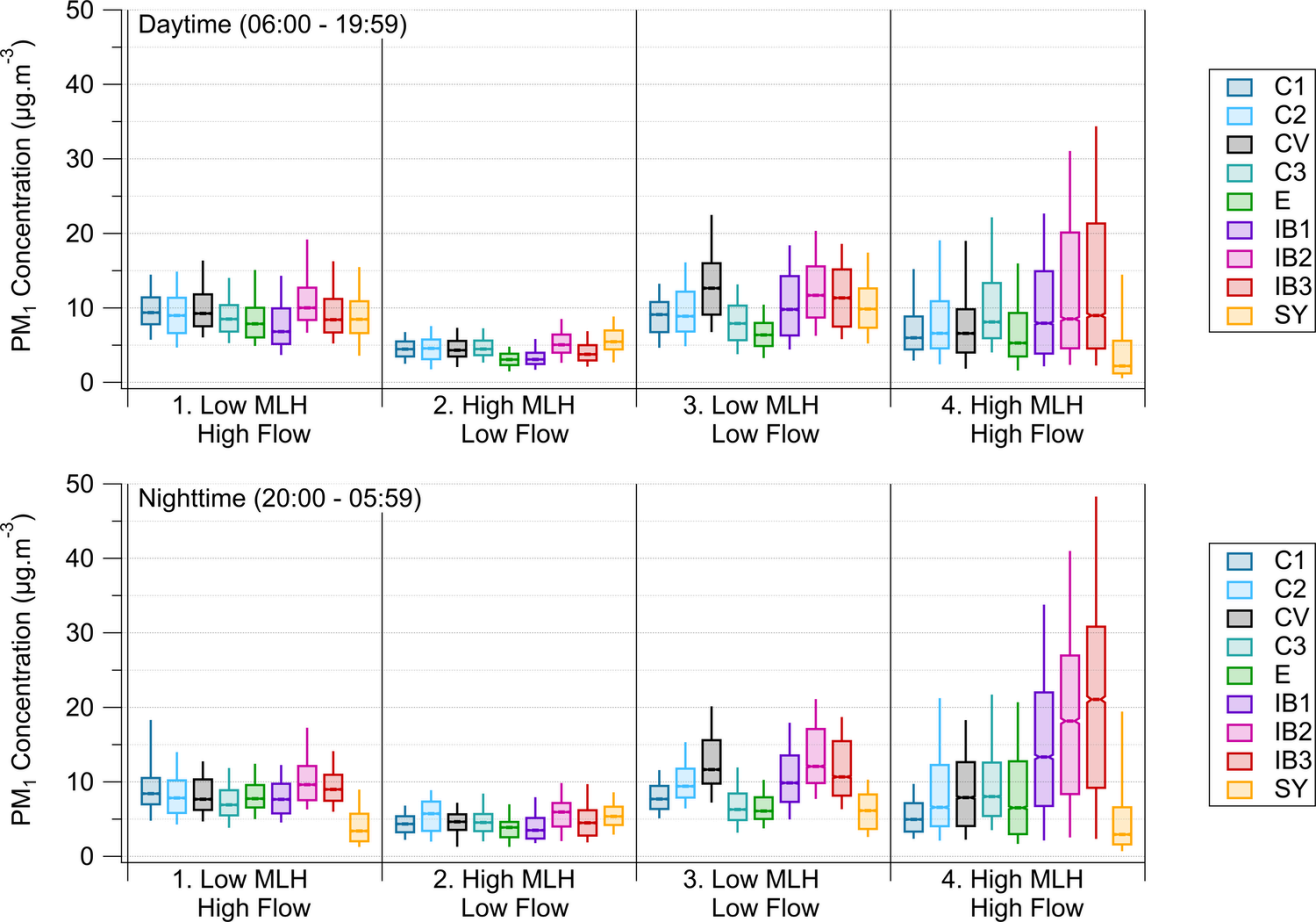
Boxplot summarizing daytime (top) and nighttime (bottom) PM_1_ observations across all sites for each defined period. Horizontal lines represent median concentrations, with the notch symbolizing their 95% confidence interval. Lower and upper whiskers denote the 9th and 91st percentiles, respectively. Outliers are not included.

The observed PM_1_ variability is used here to investigate the hypothesis that the polluted TR is an identifiable source of VOCs acting as precursors for SOA. While it is recognized that the TR may also serve as a source of primary particles, particularly at the turbulent hotspot, this work focuses on the hypothesis that river-to-air transfer of gaseous compounds—driven by the increased concentrations of contaminants and turbulence—contributes to secondary aerosol precursors and explains the PM_1_ observations. Differences and similarities in the spatial-temporal distribution of PM_1_ across the nine sites were analyzed, providing evidence that supports the hypothesis and yielding insights into other sources contributing to PM_1_ levels in the region. The role of meteorology in influencing the observed trends is also elucidated. Low transboundary TR flow conditions (Periods 2 and 3) are addressed first in the discussion, followed by high transboundary TR flow conditions (Periods 1 and 4). This is followed by analysis and discussion of apparent discrepancies with the hypothesis (Section 3.2). The discussion concludes with an analysis of the time-resolved diurnal trends of PM_1_, PM_2.5_, and their ratio, along with additional particle sizing measurements that further support that the polluted river is a source of VOCs that act as precursors for SOA formation (Section 3.3).

#### 3.1.1 Low Transboundary TR Flows (Periods 2 and 3)

Periods 2 and 3 shared similar TR discharge rates and ambient temperatures, but Period 2 was distinguished by improved vertical mixing, as indicated by the lower d*T* estimates (Table 1). This difference translated into distinct PM_1_ levels between the two sampling intervals. The lowest mass concentrations were observed during Period 2, with daytime and nighttime medians ranging from 3.1 to 5.5 μg m^–3^ and from 3.5 to 6.0 μg m^–3^, respectively. During Period 3, daytime median concentrations varied from 6.4 to 12.7 μg m^–3^; at night, these levels ranged from 6.1 to 12.1 μg m^–3^.

The lower PM_1_ levels across all sites during Period 2 can be attributed to the expanded MLHs. During this period, the mean MLH diurnal peak was 675 ± 175 m, whereas in Period 3, this value decreased to 222 ± 64 m (Figures S16 and S17). The enhanced dispersion of ground-level pollutants at high MLHs also leads to reduced PM_1_ variability, as demonstrated by the small interquartile ranges (IQRs) of Period 2. Maximum daytime and nighttime IQRs exhibited a 3-fold and 2-fold increase, respectively, following the decrease in MLHs from Period 2 to Period 3. This relationship between MLH and PM concentrations has been extensively discussed in previous studies that elucidate the role of meteorology in regulating the distribution of atmospheric pollutants.^37,38,40–44^

Despite the low PM_1_ concentrations, the distributions of PM_1_ in Period 2 provided insights into the diversity of sources contributing to PM_1_ levels at different sites in the region. For instance, daytime concentrations were more similar among coastal sites (C1, C2, and C3_0) than among more eastern inland sites (IB1, IB2, IB3, and SY). The variety of sources contributing to PM_1_ at urban locations,^39^ compared to those at coastal areas,^50^ could explain the higher variability at the inland sites. This source distinction was also evident during Period 3, with inland sites IB1, IB2, IB3, and SY exhibiting higher concentrations and greater variability than those of coastal sites C1, C2, and C3. Medians and IQRs for inland sites ranged from 6.2 to 12.1 μg m^–3^ and 4.9 to 8.3 μg m^–3^, respectively. In contrast, the coastal sites had medians between 6.3 and 9.4 μg m^–3^ and IQRs ranging from 3.4 to 5.6 μg m^–3^.

The concentrations at the estuarine site E were the lowest in both Periods 2 and 3, influenced by dominant westerly winds (Figures S9–S12). This suggests that the Estuary is a weaker source of PM_1_ and gaseous precursors compared to oceanic and local urban sources under low transboundary flow conditions. The PM_1_ variability observed during Periods 2 and 3 is further explored in the Supporting Information (SI, Text S1). Subsequent discussion here is focused on the influence of contaminated transboundary flows on local PM_1_ concentrations.

During Period 2, prevailing daytime southwesterly winds at IB3 (Figure S9), the closest site to the TR hotspot, suggest that the polluted river affected PM_1_ levels at this location even under the reported zero transboundary flow conditions. In the SI, visual evidence of nonzero daytime discharges at the turbulent TR hotspot provides additional support for the observed trends (Figure S19). During nighttime, the PM_1_ median at site IB3 rose by 20%, while the IQR increased by nearly 60%. Although some sites also demonstrated broader distributions at night, this observation was not ubiquitous, suggesting that this site-specific PM_1_ increase was more likely due to localized diurnal differences in sources than to the decrease in MLHs after sunset. Given the typical rise in transboundary wastewater discharge rates at night (Figure S6B), the overnight increase in PM_1_ concentrations under low but persistent southwesterly winds at IB3 (Figure S10) supports the hypothesis that the TR-polluted waters influence PM_1_ levels at this location.

During Period 3, wind measurements (Figures S11 and S12) and high PM_1_ levels were consistent with the observation from Period 2 that the river affects localized PM_1_ concentrations at site IB3. However, further investigation of the daytime and nighttime distributions across all sites reveals the potential contribution of the TR hotspot to PM_1_ levels at additional locations in the IB region. For instance, the inland site SY is surrounded by highways and was predominantly influenced by vehicular emissions. At night, the median PM_1_ concentration at this station dropped by 38%, with the 25th and 75th percentiles decreasing by 51 and 34%, respectively. This reduction was particularly noteworthy given the prevalence of stagnant nighttime winds and low MLHs, conditions that would be expected to favor the accumulation of atmospheric pollutants, thus suggesting a significant reduction in traffic emissions during the nighttime. All remaining sites demonstrated negligible diurnal PM_1_ changes during Period 3. The sustained PM_1_ levels observed at the coastal sites C1, C2, C3, and CV could be attributed to nocturnal oceanic contributions.^50–52^ Based on the SY diurnal trends, vehicle emissions are not expected to be driving the elevated PM_1_ observations at the inland IB1, IB2, and IB3 sites.

During Period 2, the nightly rise in transboundary wastewater flows could be linked to the overnight increase in PM_1_ levels near the TR hotspot at site IB3. Furthermore, a recent study has demonstrated that under stagnant conditions, gaseous emissions from this hotspot can even have a regional impact on Southern California’s air quality.^20^ Adding this finding to the continued increase in transboundary discharge rates at night (Figure S6C) and the low nighttime winds during Period 3, the turbulent riverine site emerges as the most relevant source contributing to the sustained nighttime PM levels in the IB region.

#### 3.1.2 High Transboundary TR Flows (Periods 1 and 4)

While Periods 1 and 4 were both characterized by high transboundary TR flows, they exhibited notable differences. Specifically, the continuous nonzero discharge rates and consistently lower MLHs observed during Period 1 contrasted with the elevated, yet significantly variable, TR flows and MLHs of Period 4 (Figures S3, S15, and S18).

During Period 1, the mean MLH diurnal peak was 332 ± 161 m compared to the 546 ± 332 m observed during Period 4. The resulting enhancement in PM_1_ concentrations at low MLH is evidenced by the higher 25th percentiles observed during Period 1 compared to Period 4. Daytime first quartiles during Period 1 ranged from 5.0 to 8.2 μg m^–3^, while during Period 4, these concentrations varied from 1.1 to 5.8 μg m^–3^. However, despite limited vertical dispersion, Period 1 was overall associated with lower PM_1_ levels compared to Period 4. While the daytime 75th percentile peaked at 12.9 μg m^–3^ during Period 1, this value increased to 21.5 μg m^–3^ during Period 4. This significant difference was also evident at night, with the maximum third quartile in Period 4 reaching 31.0 μg m^–3^—more than double the 12.3 μg m^–3^ observed in Period 1.

The increase in the PM_1_ concentrations was particularly pronounced at the inland IB sites. Daytime 75th percentiles increased from Period 1 to Period 4 by 49.4, 57.5, and 89.2% at sites IB1, IB2, and IB3, respectively. The nighttime changes were even more prominent, with interperiod increases of more than 120% at IB1 and IB2 and nearly 180% at IB3. Sites IB1, IB2, and IB3 also recorded the highest IQRs during Period 4, measuring, respectively, 11.4, 15.8, and 17.1 μg m^–3^ during the day and 15.5, 18.9, and 21.9 μg m^–3^ at night. Notably, these values, which seemed influenced by the river flow variability, increased with decreasing distance to the TR hotspot, indicating that under high transboundary flow conditions, emissions from the turbulent riverine site might significantly affect PM_1_ levels in the IB region. While PM_1_ concentrations at SY decreased overnight under the stagnant nighttime conditions of Period 1 (Figure S8), elevated nightly PM_1_ observations persisted in IB, consistent with the hypothesis that the contaminated TR influences PM levels in the region. The factors potentially contributing to the unexpected differences in PM distributions between Period 1 and Period 4 (interperiod upward trend despite continuous nonzero transboundary flow conditions and lower MLHs predominant during Period 1) are explored in the subsequent section.

The influence of the polluted river on PM_1_ levels is further evidenced through a comparative analysis of the distributions observed at the IB coast (site C3_0/C3) and the TR Estuary (site E) under varying transboundary flow conditions. From Period 2 (low flow) to Period 4 (high flow), the PM_1_ medians at the IB coast increased by 81% during the day and 76.6% at night. At the Estuary, this rise in discharge rates under high MLHs led to daytime and nighttime medians increasing by 72.2 and 67.8%, respectively.

From Period 3 (low flow) to Period 1 (high flow), under low MLHs, IB coast PM_1_ medians increased by 7.5% during the day and 10% at night, while estuarine levels rose by 23.4 and 27.2%, respectively. In contrast, at coastal site C2, medians increased by only 1.1% during the day but decreased by nearly 17% at night. This suggests that the increased volume of polluted water crossing the border at high discharge rates, flowing through the TR Estuary, and emptying into the Pacific Ocean contributed to elevated PM_1_ levels at these locations.

In summary, the influence of TR emissions on PM concentrations was most evident closest to the riverine hotspot, even under low transboundary flow conditions. The persistent nighttime PM_1_ levels observed in the IB region, coinciding with increased flows and reductions in emissions from anthropogenic activities at night, underscore the significance of the contaminated TR waters as a source of precursors for secondary PM formation in the region.

### 3.2 Potential Factors Influencing the PM_1_ Variability Observed under High Transboundary TR Flow Conditions

In the previous section, evidence of the effect of the polluted TR on measured PM_1_ levels in the IB region was presented. During the two periods of high transboundary TR flows, the PM_1_ distributions showed significant differences. Period 4 exhibited the highest concentrations and variability compared to Period 1, despite improved vertical dispersion and the occurrence of zero-flow conditions. As detailed in Table 1 and illustrated in Figures S4 and S5, both ambient temperatures and RH decreased from Period 1 to Period 4. This section examines how distinct meteorological conditions may help elucidate the differences in PM_1_ distributions between these periods. The effect of radiation on atmospheric photochemical processes is also explored as a potential factor contributing to the observed variability.

#### 3.2.1 Temperature and Relative Humidity Effect on Phase Equilibria

A decrease in ambient temperature can increase PM concentrations by lowering the vapor pressure of organic species, which enhances gas-to-particle partitioning.^53,54^ When temperature or RH decreases, organic aerosol particles may transition to semisolid or solid states.^55,56^ For liquid particles, gas-particle equilibration time scales are governed by volatility, but for viscous particles, bulk diffusivity also plays a role.^57,58^

To investigate how the combination of decreased temperatures and RH from Period 1 to Period 4 might have contributed to the interperiod PM_1_ differences, particle phase fraction (*F*_p_) values were estimated based on thermodynamic equilibrium absorptive partitioning theory.^59^ *F*_p_ values were calculated for ten logarithmic-spaced volatility bins of saturation mass concentrations between 10^–4^ μg m^–3^ ≤ C* ≤ 10^5^ μg m^–3^ defined at 298 K, which encompass three different volatility classes: low-volatility organic compounds (LVOC), semivolatile organic compounds (SVOC), and intermediate-volatility organic compounds (IVOC).

*F*_p_ values were estimated using Period 1 PM_1_ measurements at IB3, and the minimum daytime and nighttime temperatures observed at this site during both Period 1 and Period 4. At low temperatures and RH, the slow re-evaporation and relatively quick establishment of local equilibrium between the gas phase and the near-surface particle lead to a decrease in the fraction of organic matter that is involved in the absorptive partitioning (*f*_om_). *F*_p_ values were calculated using *f*_om_ (Period 4) = *f*_om_ (Period 1), assuming negligible kinetic effects despite lower temperatures and RH during Period 4; and compared with *F*_p_ values calculated using *f*_om_ (Period 4) = 0.5*f*_om_ (Period 1), as a first approximation to account for the kinetic limitations that might affect phase partitioning during the colder and drier conditions of Period 4. Detailed methodology can be found in SI (Text S2).

Figure 4 illustrates *F*_p_ values as a function of Period 1 PM_1_ at daytime (top) and nighttime (bottom) minimum temperatures of Period 1 (19.9–19.6 °C) and Period 4 (8.6 and 8.7 °C). Circle and star markers represent *F*_p_ estimates for Period 1 and Period 4, respectively, and black markers indicate the average values. Left plots represent *F*_p_ estimates for *f*_om_ (Period 4) = *f*_om_ (Period 1), while right plots show the results for *f*_om_ (Period 4) = 0.5*f*_om_ (Period 1). A ubiquitous observation from these results is that, despite the interperiod temperature differences, species with C* ≤ 10^–2^ μg m^–3^ would be solely found in the particle phase (*F*_p_ ≈ 1) and species with C* ≥ 10^3^ μg m^–3^ in the gas phase (*F*_p_ ≈ 0). This highlights that at these volatility extremes, temperature-induced changes in vapor pressures would not be sufficient to change gas-particle partitioning. Thus, no interperiod change in PM mass is foreseen within this volatility range.

**Figure 4:**
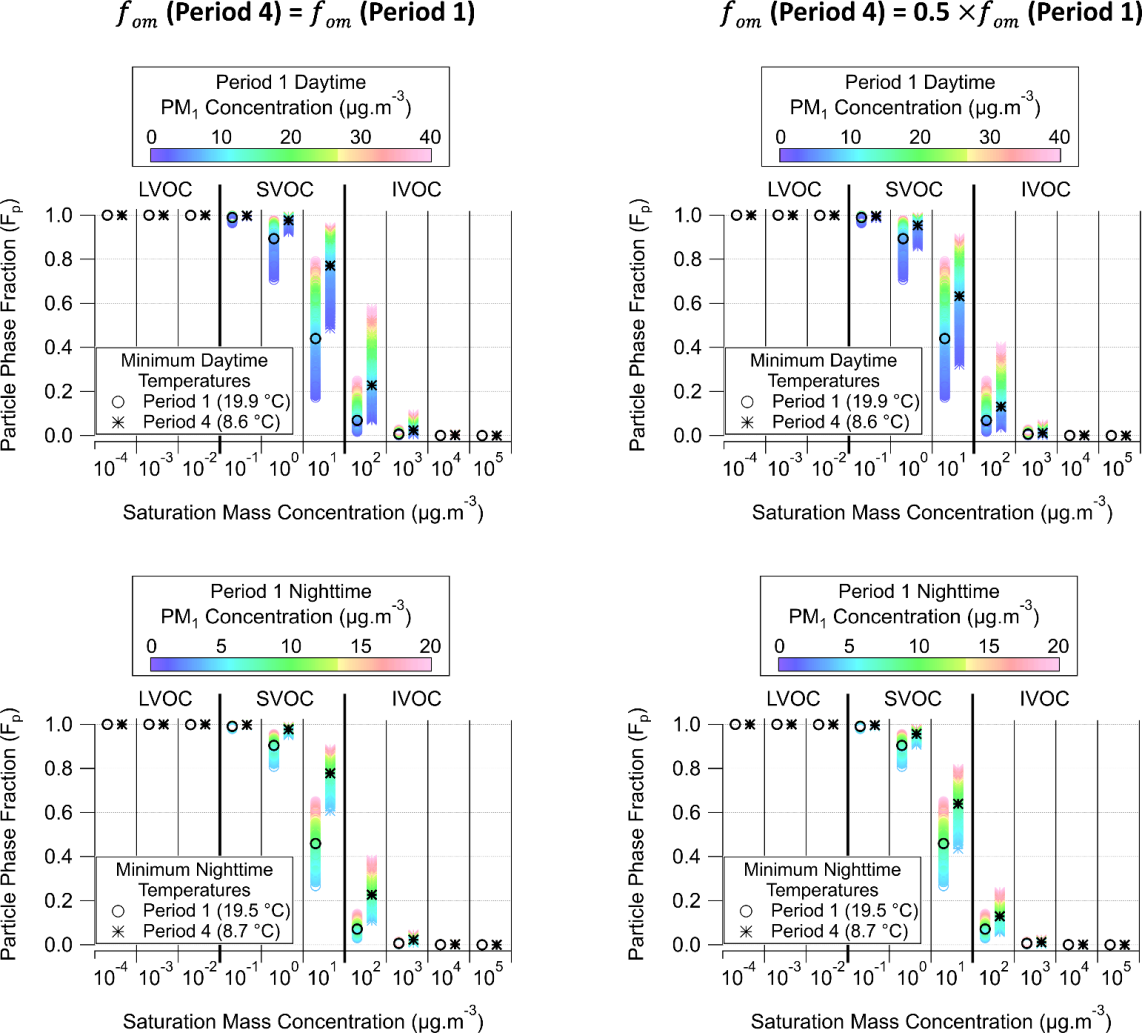
Particle phase fraction (*F*_p_) values estimated under measured Period 1 PM_1_ concentrations and measured Periods 1 and 4 minimum daytime (top) and nighttime (bottom) temperatures at site IB3. Left plots show the interperiod differences in *F*_p_ resulting solely from volatility-limited partitioning, while right plots account for kinetic limitations by reducing the fraction of organic matter that is involved in the absorptive phase partitioning (*f*_om_) by 50%.

For species within 10 μg m^–3^ ≤ C* ≤ 10^2^ μg m^–3^, higher *F*_p_ values and, consequently, increased PM mass would be expected under Period 4 cooler conditions. Assuming no kinetic limitation arises from the decrease in temperature and RH from Period 1 to Period 4, during the daytime, up to 25% of a species with C* = 10^2^ μg m^–3^ would be found in the particle phase during Period 1, increasing to nearly 60% during Period 4. Assuming a kinetic limitation, represented by a 50% reduction in *f*_om_ during Period 4, this upper bound would then be closer to 40%. Even with consideration of a kinetic limitation, the *F*_p_ limit is significantly higher in Period 4 than in Period 1.

Investigating nighttime partitioning for species with C* = 10^1^ μg m^–3^, the interperiod temperature decrease would promote a significant increase in *F*_p_ values. For volatility-limited phase partitioning (i.e., no kinetic limitation), the average *F*_p_ value in Period 4 would be close to 0.8, representing a 74% increase compared to the average *F*_p_ value in Period 1. If kinetic limitations are considered (i.e., 50% interperiod decrease in *f*_om_), the average *F*_p_ value for Period 4 would decrease to 0.64. This is higher than the average *F*_p_ of 0.45 estimated for Period 1. These results illustrate the temperature effect in lowering the vapor pressure of I/SVOC species, enhancing condensation, and likely contributing to the elevated PM_1_ concentrations during Period 4 relative to Period 1.

To isolate the effect of temperature on the observed PM levels during Period 4, an additional sampling period (Period 5) was analyzed. Table S2 summarizes the meteorological and flow conditions observed during Period 5, and Figure S20 illustrates the interperiod comparison showing that both daytime and nighttime PM_1_ concentrations decreased in Period 5 at sites IB1, IB2, and IB3 with decreases in transboundary flows. These results elucidate the significance of temperature and RH on influencing PM_1_ mass and support the hypothesis that the TR is an important source of gaseous precursors, which increase with higher transboundary flows.

#### 3.2.2 Radiation Effect on Atmospheric Photochemical Processes

SOA yields and lifetime are significantly affected by solar irradiance.^42^ Photochemical processes contribute to the total SOA budget by promoting OH-oxidative functionalization of organic molecules, photosensitized SOA oxidation, and the photolytic degradation of gaseous precursors and condensed species that absorb at actinic wavelengths.^60–63^ Modeling results and laboratory observations suggest that SOA exposure to ultraviolet (UV) radiation has the potential to control SOA atmospheric budget due to the fast photolysis mass loss rates at relevant atmospheric time scales.^42,64^ However, the total photolytic mass loss depends on the fraction of photoactive and nonphotoactive molecules initially present in the SOA particles and further produced through combined photodegradation and relevant oxidative processes.^65^

Photochemically driven SOA degradation is viewed as a more significant factor affecting PM_1_ levels in the IB region than OH-driven SOA formation because of lower precursor concentrations during the daytime. In Section 3.3.1, it is shown that PM levels at site IB3 actually decrease during peak photochemical hours. Consequently, radiative forcing is analyzed herein with regard to its capacity to reduce SOA mass.

For a given SOA system, photolytic SOA losses are described by a first-order reaction with respect to the spectral actinic flux.^42,65^ To assess whether variations in actinic fluxes may have influenced the differences in PM_1_ concentrations under high transboundary flows in Periods 1 and 4, it was assumed that the SOA composition was similar during both periods. The Tropospheric Ultraviolet and Visible (TUV) Radiation Model^66^ was used to estimate the UV actinic flux at 10 m above ground during Periods 1 and 4 at IB3 coordinates. Model inputs are summarized in Table S3, and the results are displayed in Figure 5.

**Figure 5:**
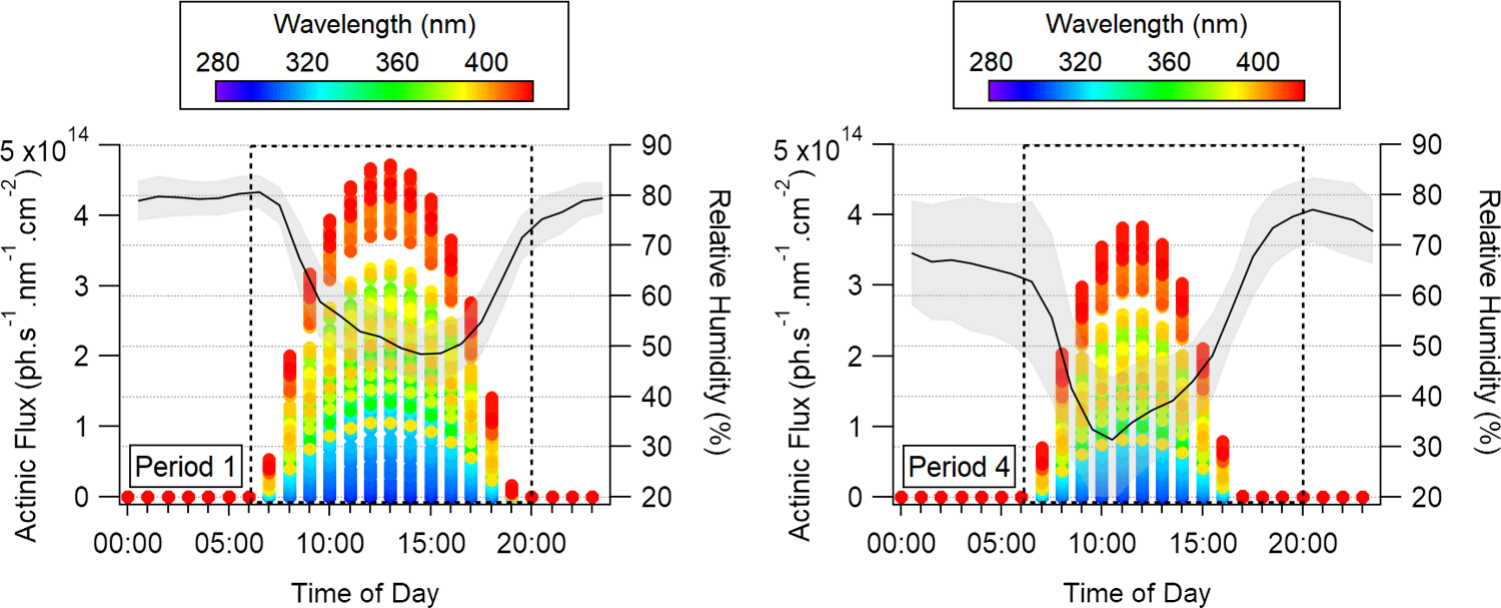
Modeled actinic fluxes at site IB3 during Period 1 (left) and Period 4 (right). Dashed boxes represent the defined daytime window. Measured RH diurnal profiles are plotted on the right axis, with shaded areas representing one standard deviation of the mean.

Higher actinic fluxes were observed during Period 1 in comparison with Period 4. Additionally, earlier sunsets during Period 4 resulted in a 2 h day^–1^ decrease in solar irradiance within the considered daytime window (dashed box), thus preventing photochemical processes from influencing the atmospheric SOA budget during these dark hours. As photolysis frequencies increase at higher photon fluxes and longer exposure times, SOA formed during Period 1 should have experienced larger photolysis-driven mass losses, resulting in lower PM_1_ levels compared to those from Period 4. The higher RH in Period 1 might have enhanced the photolytic SOA mass losses, as significantly faster photolysis rates have been observed in less-viscous particles due to efficient kinetics within the SOA matrix.^63^

### 3.3 Additional Evidence Linking the Tijuana River Gases to PM_1_ Levels via Secondary Aerosol Formation

#### 3.3.1 PM_1_ and PM_2.5_ Time-Resolved Diurnal Profiles

Figure 6 illustrates the time-resolved diurnal profiles for PM_1_ and PM_2.5_ at sites SY (traffic-dominated) and IB3 (TR-dominated) during the four main periods considered in this study. The PM_1_ to PM_2.5_ ratio (herein referred to as the PM ratio) is also plotted to help elucidate the effect of meteorology and atmospheric processing on the transformation and fate of these pollutants. One consistent finding across all periods and both sites is that the diurnal variability of PM_1_ and PM_2.5_ is highly correlated. Correlation coefficients ranged from 0.77 to 0.99 at site SY and from 0.84 to 0.99 at site IB3, indicating that PM_1_ constitutes a large fraction of PM_2.5_ in both locations.

**Figure 6:**
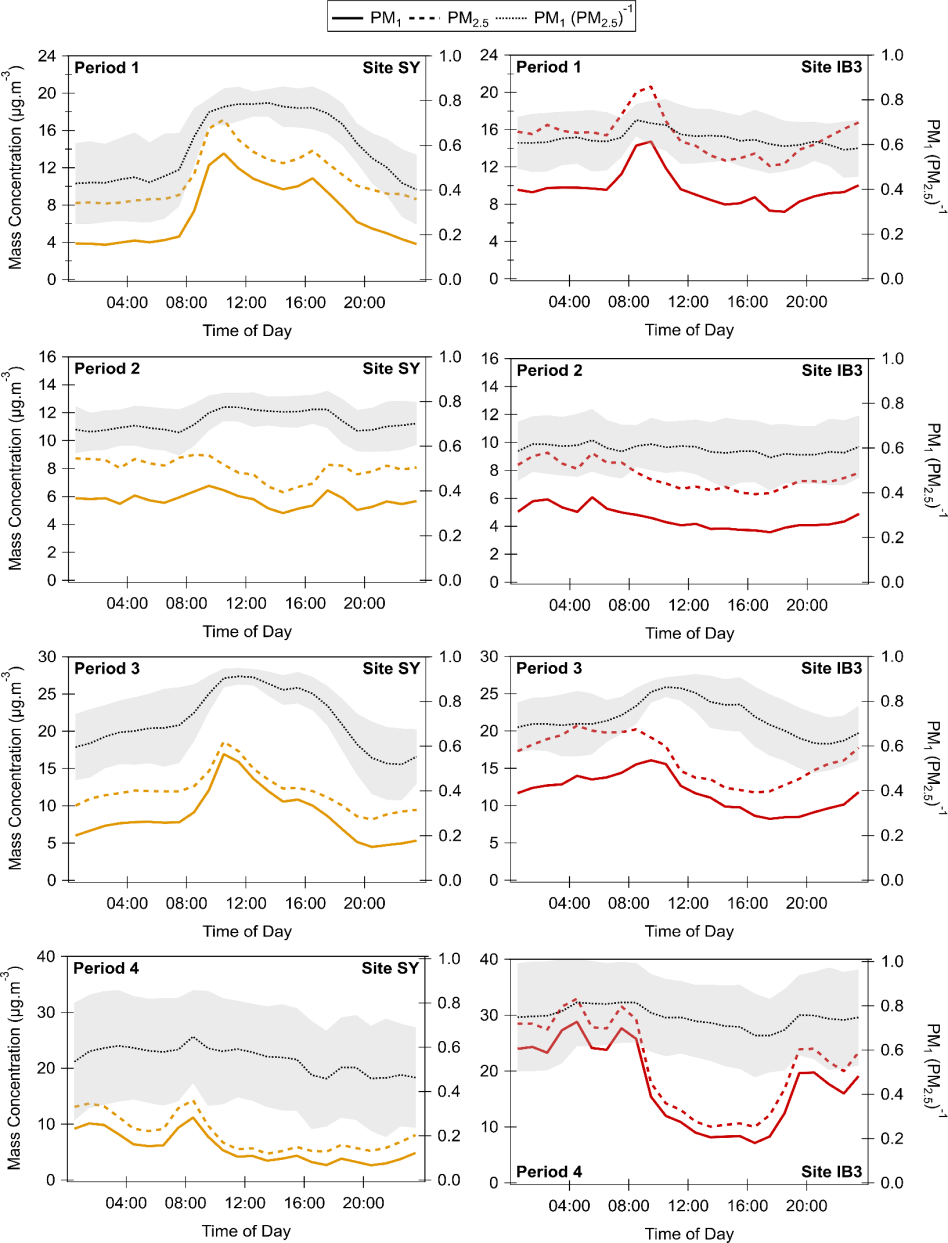
Diurnal profiles of PM_1_ (solid line), PM_2.5_ (long-dash line), and their ratio (short-dash line) for sites SY (left) and IB3 (right) for the four main periods considered in this study (refer to Table 1). Shaded region represents one standard deviation of the PM ratio mean.

During Period 1, a bimodal PM peak was observed at SY, characterized by two distinct peaks, one in the morning and one in the early evening hours. This reflects daily commuting patterns and suggests vehicular emissions as an SOA source at this location. SOA has been found to exceed primary PM emissions for light-duty gasoline vehicles.^67^ The PM ratio was consistently high between and inclusive of these peaks, revealing the higher daytime PM_1_ contribution to total PM_2.5_. This is consistent with efficient SOA formation from motor vehicle exhaust emissions^68^ and increased SOA formation during peak photochemistry.

At IB3, the PM ratios were consistently high, with daytime values from 0.59 ± 0.11 to 0.71 ± 0.07 and nighttime values from 0.58 ± 0.12 to 0.63 ± 0.12. These high PM ratios highlight the persistent contribution of submicrometer particles to the total PM_2.5_. The consistent daytime-to-nighttime PM ratios observed at site IB3 can be explained by the diurnal variability in source strength and meteorological conditions during Period 1. The NO_3_ radical (nighttime oxidant) is more selective than OH (daytime oxidant),^69^ and thus promotes the overnight accumulation of many nightly emitted VOCs at lower nighttime MLHs. As the sun rises and OH concentrations increase, these VOCs are rapidly oxidized, increasing the PM_1_ due to SOA formation around 08:00. This also increases the PM ratio. The PM ratio then quickly stabilizes in response to lower daytime flows and expanded MLHs, which decrease VOC concentrations and reduce SOA yields.

During Period 2, despite more atmospheric mixing, photochemical transformation of vehicular emissions at site SY was still significant, as shown by the high daytime PM ratios (≥0.67 ± 0.09). Conversely, the improved vertical dispersion during both the day and night prevented the overnight accumulation of riverine gaseous SOA precursors at site IB3, leading to the absence of a morning increase in SOA and a relatively stable PM ratio, ranging from 0.56 ± 0.15 to 0.63 ± 0.14.

During Period 3, the minimal vertical dispersion contributed to increased concentrations of ground-level emissions, thereby promoting SOA formation. Indeed, site SY had the highest PM ratios during the day, ranging from 0.61 ± 0.14 to 0.91 ± 0.04. At site IB3, daytime ratios varied from 0.63 ± 0.13 to 0.86 ± 0.04 and demonstrated a more gradual reduction when compared to Period 1. This is because, despite the decrease in riverine emissions during the day, SOA precursors and their products remained concentrated near the surface and were more available for oxidation and SOA formation.

During Period 4, high MLHs effectively diluted surface emissions, leading to a decrease in SOA formation at site SY. This is indicated by the lowest daytime PM ratios recorded at this location throughout the four main periods examined in this study (0.46 ± 0.22 to 0.65 ± 0.21). At site IB3, PM ratios remained high, with daytime values ranging from 0.66 ± 0.16 to 0.81 ± 0.18, and nighttime values from 0.74 ± 0.22 to 0.82 ± 0.18.

If the contribution of the TR to PM levels at site IB3 was largely through aerosolization, one would expect PM diurnal profiles to mirror the TR flow diurnal profiles (Figure S6). However, correlation coefficients between PM_1_ concentrations and transboundary flows were below 0.4, indicating no clear temporal correlation between the two variables. Additionally, the PM_1_ increase at IB3, corresponding to OH-initiated oxidation during Periods 1 and 3, would not have been observed if these submicrometer particles were mainly formed by the physical process of bubble bursting. Existing literature supports that SOA formation predominantly leads to mass growth of submicron particles;^70^ therefore, the high PM ratios observed at IB3, even under diluted atmospheric conditions, reinforce the significance of the TR as an important source of gaseous precursors that contribute to increases in PM_1_ concentrations through SOA formation.

#### 3.3.2 PM_1_ Size Discretization

In addition to mass concentrations, QuantAQs provided particle size distribution data from 0.35 to 40.0 μm, subdivided into 24 bins. The PM_1_ measurements encompass three size bins: bin zero, corresponding to the total number of particles with diameters between 0.35 and 0.46 μm; bin one, corresponding to 0.46 to 0.66 μm particles; and bin two, which includes 0.66 to 1.0 μm particles. Since Period 4 was characterized by the highest PM_1_ levels associated with high transboundary TR flow rates, the size-dependent increase in PM_1_ concentrations during this period was investigated at sites SY (traffic-dominated) and IB3 (TR-dominated) using the following methodology.

For each site, data points were filtered based on the daytime and nighttime classification used in this paper. Bin zero (0.35–0.46 μm particles) was normalized on a 0–1 scale and averaged as a function of time (hourly) across Period 4 sampling days. Then, bins one and two were combined into a single bin representing 0.46–1.0 μm particles and then processed akin to bin zero. Figure 7 illustrates bin zero plotted against the combined bins one and two at sites SY (triangle markers) and IB3 (circle markers) during both the daytime (left) and nighttime (right). The markers are color-coded according to the time of day, while the solid lines denote the linear fits for each data set.

**Figure 7:**
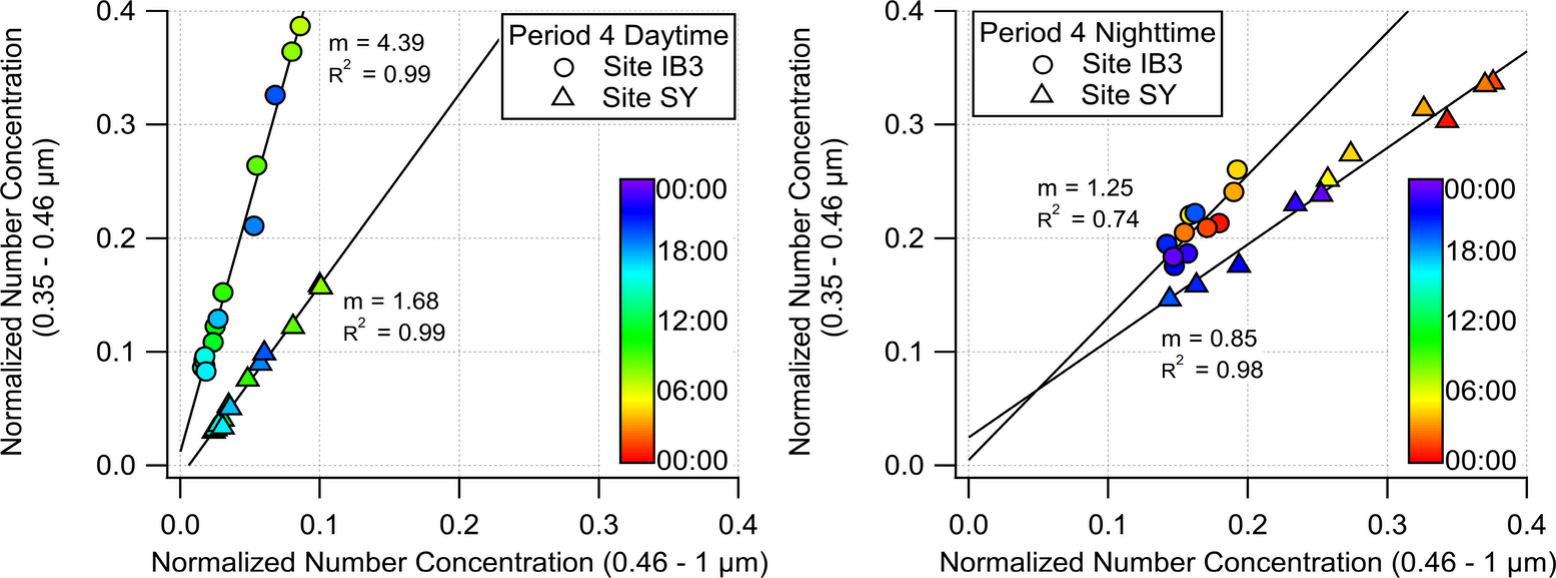
Daytime (left) and nighttime (right) normalized PM_1_ size distributions from QuantAQ measurements at sites SY (triangle-markers) and IB3 (circle-markers) during Period 4.

The daytime slope at site SY (*m* = 1.68) highlights the size-dependent increase in PM_1_ levels, largely influenced by heavy-traffic highway gaseous and particle emissions. The daytime slope at site IB3 (*m* = 4.39) was nearly three times higher than that observed at SY, indicating that PM_1_ levels at IB3 are primarily influenced by the faster increase in the number of smaller-sized particles. At both sites, daytime observations showed steeper slopes compared to nighttime, indicating a faster increase in the smaller size bin (0.35–0.46 μm particles) during the day. This could be a result of an increase in primary particle and precursor emissions during the day and faster OH-initiated oxidation, contrasting with a decrease in these emissions during the night and generally slower nocturnal NO_3_ chemistry.^69^ Indeed, the nighttime slope at SY fell below one (*m* = 0.85), indicating an increase in larger-sized particles (>0.46 μm) during the night at this location. However, the nighttime slope at site IB3 was above one (*m* = 1.25), indicating an increase in the smaller-sized particles (<0.46 μm). This observation is consistent with the effective oxidation of gaseous precursors^70^ emitted from the TR, and provides a link between the abundance of VOCs emitted from the turbulent TR hotspot^20^ and the measured PM_1_ concentrations.

Measurements obtained from the QuantAQ MODULAIR monitor and the research-grade OPS were also compared. PM_1_ estimates from the MODULAIR (OPC only) and the OPS at RH levels below 70% at site IB3 during Period 4 are shown in Figure 8 (top row). This RH threshold was chosen based on the analysis in Section 2.2.1. Here, it is reiterated through Period 4-only results that the low-cost OPC is robust, as it shows good agreement with the research-grade OPS at low-to-moderate RH levels.

**Figure 8:**
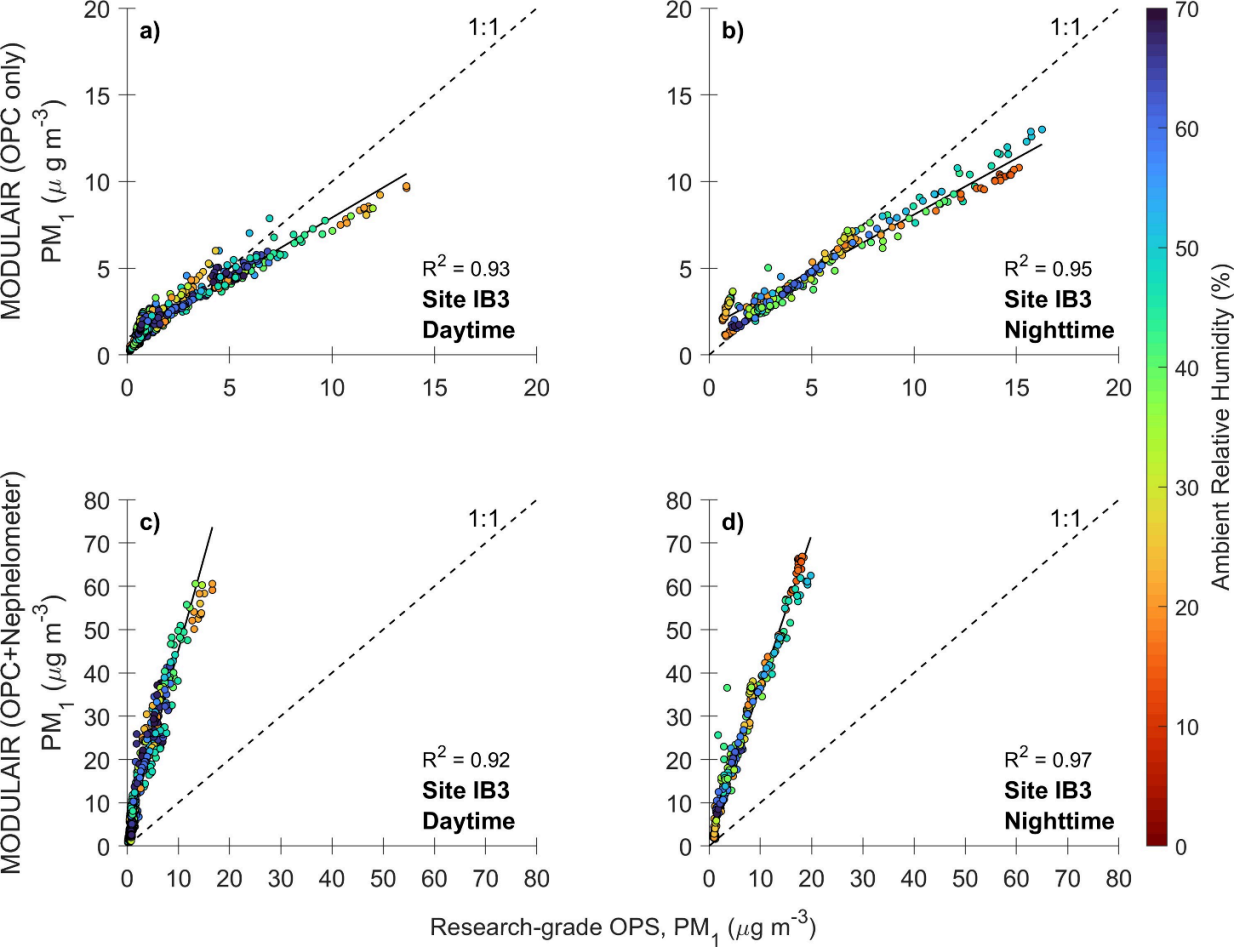
RH-dependent daytime and nighttime linear regression plot comparing the OPS 3330 to the MODULAIR (OPC; top row) and MODULAIR (OPC + nephelometer; bottom row) at site IB3 during Period 4 for ambient RH below 70%.

When adding the nephelometer data from the MODULAIR (OPC + nephelometer) and comparing with the OPS (Figure 8, bottom row), the low-cost sensor shows substantially higher PM_1_ estimates compared with the research-grade OPS. This discrepancy can be attributed to the limitations of the OPS in measuring particles smaller than 0.3 μm, which the nephelometer effectively captures. Therefore, the high contribution of sub-300 nm particles to total PM_1_ mass demonstrated by the MODULAIR’s nephelometer measurements supports the hypothesis that secondary chemistry plays a significant role in the PM_1_ observations.

### 3.4 Implications

This study elucidates the significance of intercompartmental transfer (river-to-air) of pollutant precursors from the contaminated TR in San Diego County and its contribution to poor air quality. The integrated investigation of the spatial-temporal variability of PM_1_, transboundary TR flows, and local meteorological conditions suggests that contaminated water bodies can significantly influence PM levels in a coastal urban setting. Local PM_1_ levels were lowest under low transboundary river flow conditions and effective vertical dispersion; however, even under such conditions, the influence of the contaminated TR on PM_1_ concentrations could still be observed near the turbulent riverine hotspot. A decrease in MLH limited the dispersion of ground-level pollutants, leading to widespread increases in PM_1_ levels throughout the region, with inland sites exhibiting higher concentrations and greater variability than those of coastal sites. An analysis of diurnal trends across all sites revealed the TR as the most relevant source contributing to sustained nighttime PM_1_ levels in the region, even during low river flow conditions.

The spatial correlation between the PM_1_ distributions and the riverine hotspot during high transboundary flows highlights the substantial effect of the polluted TR on local PM_1_ levels. The highest concentrations observed at night coincided with peaks in wastewater flows, further emphasizing the significance of the intercompartmental pollution transfer in the region. Although variations in meteorological conditions (i.e., ambient temperature, RH, and actinic fluxes) throughout the study period are acknowledged to have influenced the observed PM_1_ levels, further analysis isolating the convoluted variables (i.e., transboundary flows and meteorology) suggested that river-to-air emission enhancement due to increased wastewater discharge rates contributes to increases in near-source PM_1_ concentrations. The higher contribution of smaller particles to the PM_1_ mass indicated by measurements from both low-cost and reference method equivalent instrumentation specifically demonstrates that secondary chemistry fueled by riverine emissions largely drives the PM_1_ observations. This is further corroborated by the sustained high PM_1_ to PM_2.5_ ratios (≥0.56 ± 0.15), indicating that submicrometer particles dominate the PM levels. The increase in PM_1_ concentrations at the estuary and coastal sites in Imperial Beach, coinciding with increasing transboundary flows, challenges the conventional perspective that this pollution is too diluted to impact coastal air quality. Further work is needed to quantitatively address the extent to which TR gaseous emissions contribute to SOA formation and PM pollution in the region.

This work brings attention to a coastal urban region that has endured the consequences of the persistent contamination of the TR that has accompanied economic and industrial development of the Mexico-US border. However, the implications of these findings expand beyond San Diego County, California. Due to industrial pollution discharges, the Passaic River in New Jersey and the Calcasieu River in Louisiana are, together with the TR, listed among America’s ten most endangered rivers (2025).^71^ Moreover, research indicates that over 40% of the world’s freshwater ecosystems are severely polluted.^72^ Thus, the connection between contaminated TR and urban airborne pollution highlighted by this study emphasizes the importance of addressing the potential for increases in PM_1_ levels in various urban and industrialized regions adjacent to water bodies worldwide.

The US EPA has recently strengthened the National Ambient Air Quality Standards for Particulate Matter (PM NAAQS) by setting the level of the primary annual PM_2.5_ standard at 9.0 μg m^–3^ while maintaining the primary 24 h PM_2.5_ standard at the level of 35 μg m^–3^.^73^ Although PM_2.5_ standards encompass PM_1_ measurements, evidence suggests that exposure to PM_1_ may be more detrimental to human health.^74,75^ Smaller particles can penetrate deeper into the lungs and sometimes infiltrate cellular membranes completely,^76^ resulting in a greater potential for adverse health outcomes.^77^ Short-term PM_1_ exposure has been shown to increase the risk of cardiovascular and respiratory mortality,^78^ while long-term exposure has been linked to an increase of 1.7–22% in all-cause mortality.^79^

PM_1_ also plays a significant role in regulating the regional and global climate by influencing Earth’s radiative balance through direct (scattering and absorption) and indirect (cloud formation and precipitation) effects. Recently, terrestrial nutrient inputs were found to enhance marine VOC emissions,^80^ while it has been demonstrated that secondary marine aerosol plays a dominant role over primary sea spray aerosol in cloud formation.^81^ The accelerated development of the Anthropocene is bound to culminate in greater pollutant discharge into Earth’s natural systems. About 80% of the world’s wastewater remains untreated,^82^ yet contaminated water bodies are currently overlooked as emission sources in existing climate and air quality models. As rising temperatures exacerbate evaporative emissions from polluted waterways,^83^ representing intercompartmental pollution transfer becomes crucial for mitigating the current crisis and avoiding future impacts.

## Supplementary Material



## Notes

Further discussion on PM_1_ variability, calculation methods, and supplementary tables and figures, including meteorological data and TR photographs (PDF)
