# Supplementary material for: Contaminated Tijuana River Contributes to Regional Particulate Matter (PM) Levels Highlighting Overlooked Water–Air Transfer of Gaseous PM Precursors
Source: Environ Sci Technol. 2025 Dec 16;59(51):27903–17. doi: 10.1021/acs.est.5c08369 (PMC12756918; doi:10.1021/acs.est.5c08369)
Supplement: Supplementary file 1 [file es5c08369_si_001.pdf]

Supporting Information for:

Contaminated Tijuana River Contributes to Regional Particulate Matter (PM) Levels Highlighting Overlooked Water-Air Transfer of Gaseous PM Precursors

Karolina Cysneiros de Carvalho[1], Kelley C. Barsanti[2], Justin D. Hamlin[3], Kimberly A. Prather[†3], William C. Porter[1]*

[1]Department of Environmental Sciences, University of California Riverside, Riverside, California, 92521, United States.

[2]Atmospheric Chemistry Observations & Modeling Laboratory, NSF National Center for Atmospheric Research, Boulder, Colorado, 80305, United States.

[3]Department of Chemistry and Biochemistry, University of California San Diego, La Jolla, California, 92093, United States.

[†]Scripps Institution of Oceanography, University of California San Diego, La Jolla, California, 92037, United States.

*Corresponding Author: Kelley C. Barsanti (barsanti@ucar.edu)

Contents of this file:

Number of Text: 2 (S1-S2)

Number of Figures: 20 (S1-S20)

Number of Tables: 4 (S1-S4)

Text S1. Additional Discussion on the PM_1 Spatial and Temporal Variability

Although the distinction between urban and coastal sources influencing PM_1 levels in the region was evident during Periods 2 and 3 (as discussed in Section 3.1), significant trends became apparent due to the differing MLHs observed during these two periods. For instance, in Period 2, the ocean-influenced sites C1, C2, C3_0, and CV exhibited similar concentrations, with medians fluctuating within a narrow range of 4.3 to 5.7 μg m^{-3}. However, during Period 3, these levels increased and varied across a wider range of 6.3 to 12.7 μg m^{-3}, primarily driven by the distributions observed at C3 and CV.

During Period 3, site C3 demonstrated the lowest median concentration and 25th and 75th percentiles among the sites mentioned above. This could be attributed to the C3 sensor being deployed on a beachfront property, which reduces the influence of non-oceanic emissions on its PM_1 measurements. In contrast, sites C1, C2, and CV were located in areas accessible to vehicles, making them susceptible to engine combustion emissions. These differences were not observed in Period 2 as a result of the effective pollutant dispersion, which lessens the impact of non-dominant sources, such as vehicle-related emissions at coastal locations.

The residential site CV, located east of San Diego Bay and Highway I-5, exhibited the highest daytime PM_1 median among all monitored locations during Period 3. This value was equal to 12.7 μg m^{-3}, which is approximately 43% higher than that of C2, the coastal site on the western side of the bay, and reflects a 28% increase when compared to the value observed at SY, the urban site predominantly influenced by highway-traffic pollutants. Thus, suggesting that oceanic and urban emissions contributed to the observed PM levels in CV. While wind observations were unavailable at this station, measurements obtained at C2 indicate that northwesterly winds drive

the distribution of pollutants at this latitude, providing additional support for the influence of both sources on CV pollutant levels under low MLHs.

The above analysis proposes that during Period 3, PM_1 concentrations were primarily driven by nearby sources, with the observed levels enhanced due to low MLHs. This is further corroborated when considering the inland distributions. During Period 2, site SY exhibited higher PM_1 concentrations compared to sites IB1 and IB3, implying that within the small suburban region encompassing the IB study area, the impact of local sources is comparatively minor relative to the heavy-traffic highway emissions that dominate SY levels. Nevertheless, during Period 3, PM_1 concentrations at the IB sites surpassed those recorded at SY, thereby underscoring how the reduction in MLHs limits the dispersion of ground-level pollutants.

Text S2. Particle Phase Fraction Estimations

Volatility-dependent particle phase fraction (F_p) estimations were performed considering the thermodynamic equilibrium absorptive partitioning theory.[1] F_p values were calculated for ten logarithmic-spaced volatility bins of saturation mass concentrations between $10^{-4} \leq C^* \leq 10^5$ µg m^{-3} defined at 298 K, and encompassing three different volatility classes: low-volatility organic compounds (LVOC), semi-volatile organic compounds (SVOC), and intermediate-volatility organic compounds (IVOC). For each C* bin (in µg m^{-3}), the corresponding liquid (or sub-cooled) vapor pressure (p_L°) at the reference temperature of 298 K was calculated using Equation S1.

$$p_L^o(T_{ref}) = C^* \frac{760RT_{ref}}{10^6 MW} \quad \mathbf{(S1)}$$

Where $p_L°(T_{ref})$ is given in Torr, T_{ref} is the reference temperature in K; MW is the molecular weight in g mol^{-1} of the corresponding volatility bin; R (8.2×10^{-5} m^3 atm mol^{-1} K^{-1}) is the ideal gas constant; 760 is a pressure conversion factor; and 10^6 is a mass conversion factor.

Then, Equation S2 was used to extrapolate the reference vapor pressures to each temperature of interest (i.e., Period 1 and Period 4 observations at site IB3).

$$ln\frac{p_L^o(T)}{p_L^o(T_{ref})} = -\frac{\Delta H_{vap}(T_{ref})}{R}\left(\frac{1}{T} - \frac{1}{T_{ref}}\right) \quad \textbf{(S2)}$$

Where $p_L°(T)$ is the vapor pressure in Torr at the temperature of interest; $p_L°(T_{ref})$ is the calculated vapor pressure in Torr of the respective volatility bin; R (8.314 J mol^{-1} K^{-1}) is the ideal gas constant; $\Delta H_{vap}(T_{ref})$ is the enthalpy of vaporization in J mol^{-1} of the respective volatility bin; T is the temperature of interest in K; and T_{ref} is the reference temperature in K.

The gas-particle partitioning constant (K_p) defined by Equation S3 was then used to estimate F_p values by employing Equation S4.

$$K_p = \frac{f_{om}760RT}{MW_{om}\zeta p_L^o 10^6} \quad \textbf{(S3)} \qquad F_p = \frac{K_p PM}{K_p PM + 1} \quad \textbf{(S4)}$$

Where K_p is the gas-particle partitioning constant in m^3 µg^{-1}; f_{om} is the organic fraction of the total particle mass; R (8.2×10^{-5} m^3 atm mol^{-1} K^{-1}) is the ideal gas constant; T is the temperature of interest in K; MW_{om} is the average particle-phase organic molecular weight in g mol^{-1}; ζ is the activity coefficient of the organic mixture; $p_L°$ is the vapor pressure in Torr at the temperature of interest; 760 is a pressure conversion factor; 10^6 is a mass conversion factor; and PM is the ambient total particulate matter mass concentration in µg m^{-3}.

In this work, the molecular weight corresponding to each volatility bin was assigned from the mid-point of molecular corridors that represent the relationship between molar mass and volatility at 298 K for oxidation products of anthropogenic and biogenic precursors.[2] The enthalpy of vaporization at 298 K for $C^* = 10^0$ μg.m^{-3} was assumed to be 100 kJ mol^{-1} increasing (or decreasing) by 5.8 kJ mol^{-1} for each lower (or higher) logarithmic-spaced C* bin.[3] Table S4 summarizes these properties. F_p values were evaluated using PM_1 concentrations from Period 1 observations at site IB3. A MW_{om} value of 237.5 g mol^{-1} was used for the K_p calculations (averaged across the SVOC range), and 50% of the particle mass was assumed to be composed of organic material[4] for both Period 1 and Period 4 calculations. F_p was additionally estimated at $f_{om} = 0.25$ during Period 4. Only gas-particle partitioning involving thermodynamic ideal mixtures (i.e., $\zeta = 1$) was considered.

REFERENCES

(1) Pankow, J. An Absorption Model of Gas/Particle Partitioning of Organic Compounds in the Atmosphere. *Atmospheric Environment* **1994**, *28* (2), 185–188. https://doi.org/10.1016/1352-2310(94)90093-0.

(2) Shiraiwa, M.; Berkemeier, T.; Schilling-Fahnestock, K.; Seinfeld, J. H.; Pöschl, U. Molecular Corridors and Kinetic Regimes in the Multiphase Chemical Evolution of Secondary Organic Aerosol. *Atmospheric Chemistry and Physics* **2014**, *14* (16), 8323–8341. https://doi.org/10.5194/acp-14-8323-2014.

(3) Donahue, N. M.; Robinson, A. L.; Stanier, C. O.; Pandis, S. N. Coupled Partitioning, Dilution, and Chemical Aging of Semivolatile Organics. *Environmental Science & Technology* **2006**, *40* (8), 2635–2643. https://doi.org/10.1021/es052297c.

(4) De Gouw, J.; Jimenez, J. L. Organic Aerosols in the Earth's Atmosphere. *Environmental Science & Technology* **2009**, *43* (20), 7614–7618. https://doi.org/10.1021/es9006004.

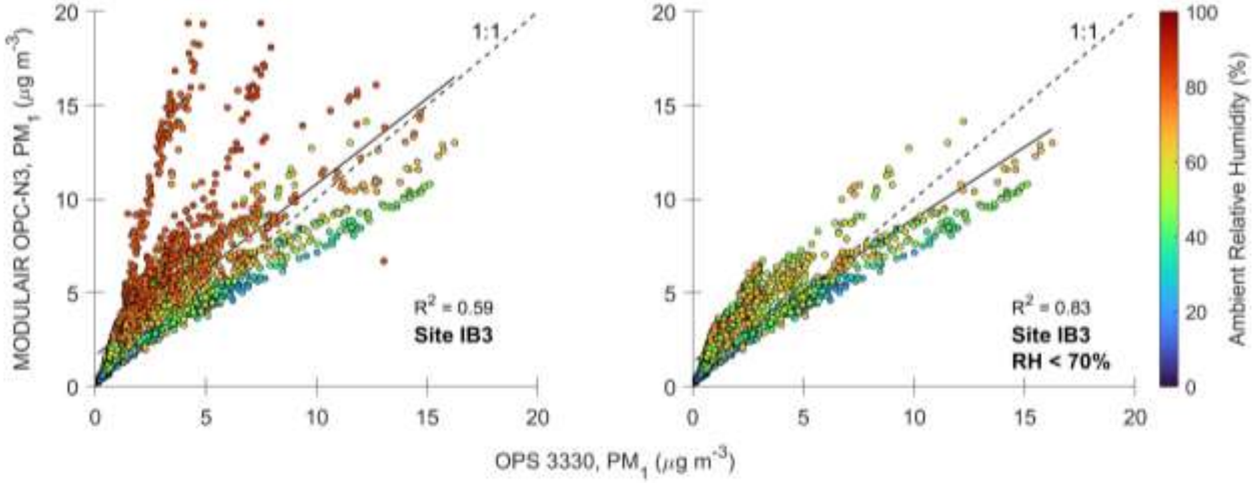

Figure S1. Linear regression plot comparing the OPS 3330 and MODULAIR^{TM} (OPC only) at site IB3, considering periodic measurements from September 1st to November 19th, 2024. The left plot shows the correlation results across all ambient RH conditions. The right plot shows the correlation results for ambient RH below 70%.

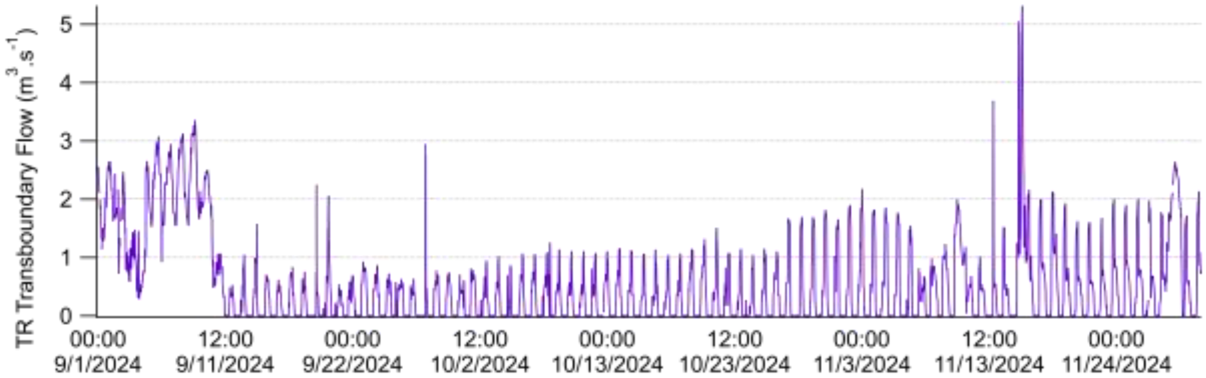

Figure S2. Linear time series of Tijuana River transboundary flow rates from September 1st to November 30th, 2024.

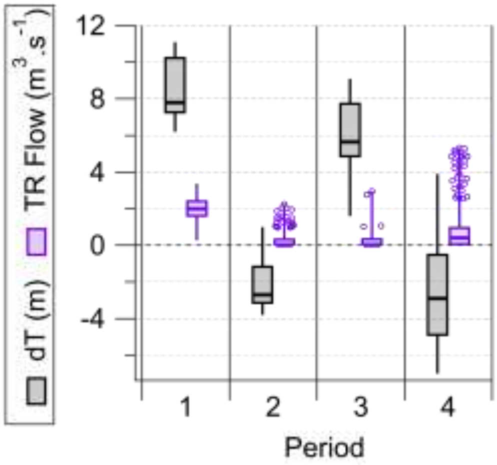

Figure S3. Boxplot comparing dT and Tijuana River transboundary flow rates across the different time periods evaluated in this study. Horizontal lines represent median values; whiskers denote maximum and minimum observations, and markers are outliers' data points.

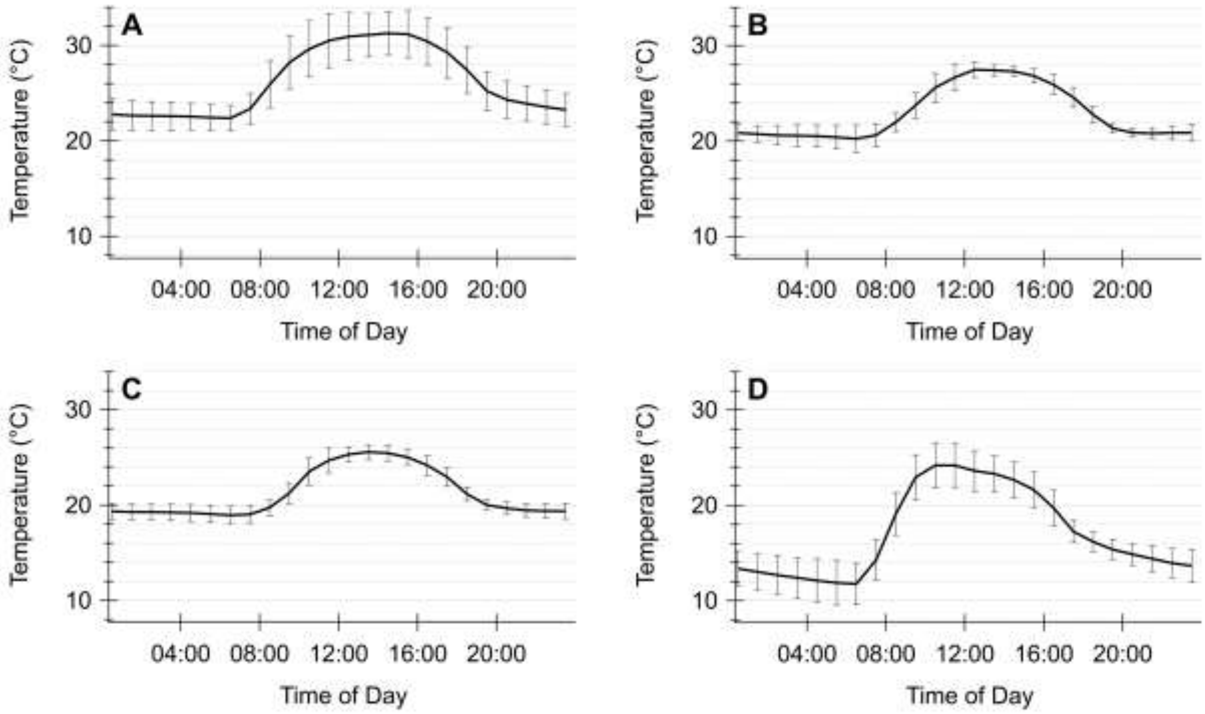

Figure S4. Diurnal profiles of ambient temperatures measured by the deployed low-cost sensors. **(A)** Period 1 (Low MLH & High Flow). **(B)** Period 2 (High MLH & Low Flow). **(C)** Period 3 (Low MLH & Low Flow). **(D)** Period 4 (High MLH & High Flow). Note: Temperature values were averaged across all sites prior to the time-averaging calculations. Error bars represent one standard deviation of the mean.

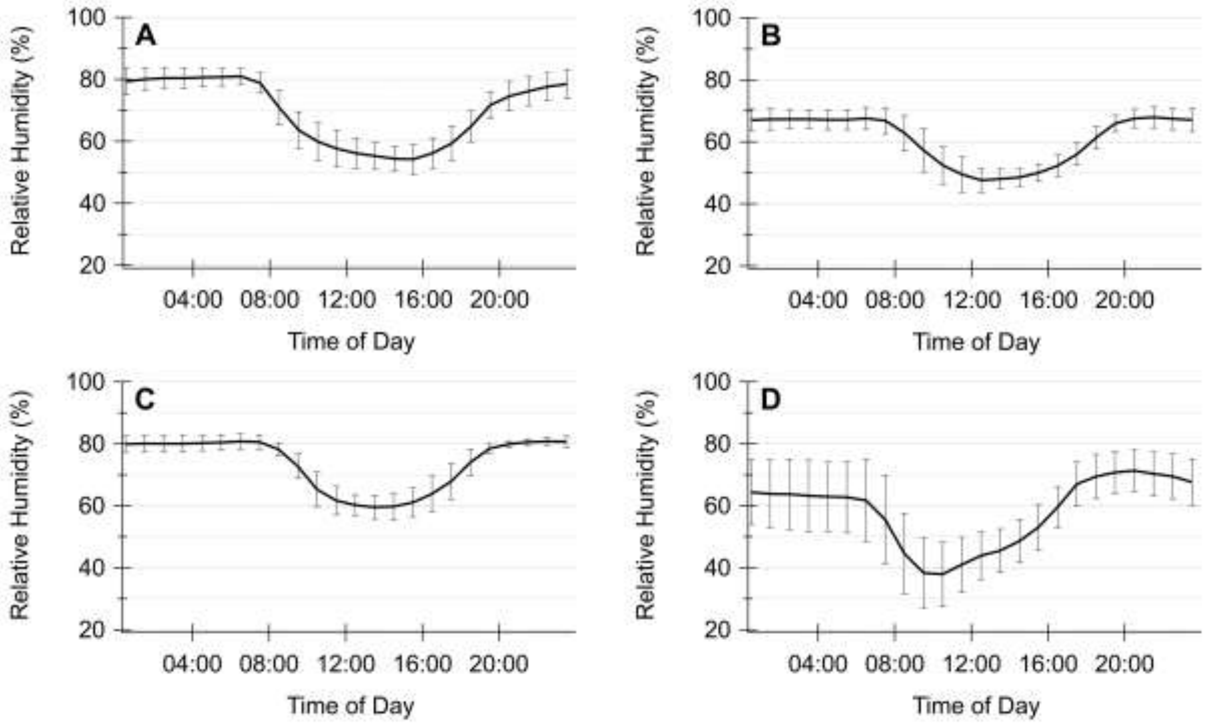

Figure S5. Diurnal profiles of ambient relative humidity (RH) measured by the deployed low-cost sensors. **(A)** Period 1 (Low MLH & High Flow). **(B)** Period 2 (High MLH & Low Flow). **(C)** Period 3 (Low MLH & Low Flow). **(D)** Period 4 (High MLH & High Flow). Note: RH values were averaged across all sites prior to the time-averaging calculations. Error bars represent one standard deviation of the mean.

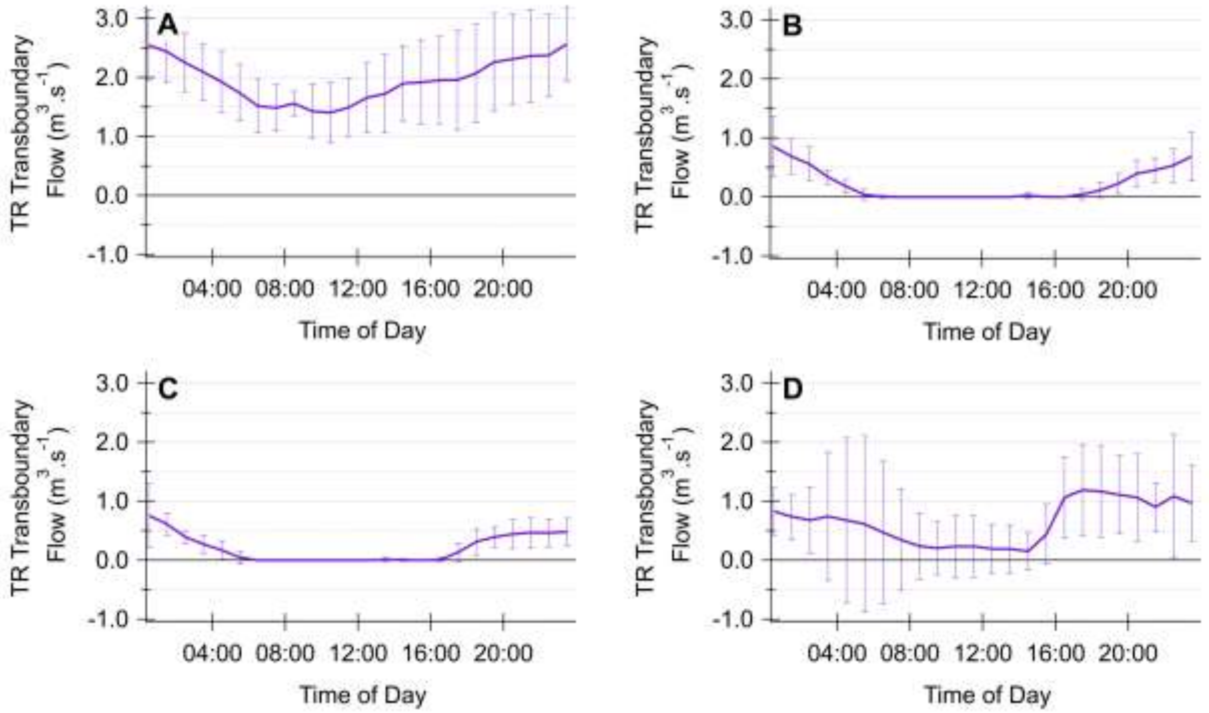

Figure S6. Diurnal profiles of Tijuana River transboundary flow rates measured at the international border. **(A)** Period 1 (Low MLH & High Flow). **(B)** Period 2 (High MLH & Low Flow). **(C)** Period 3 (Low MLH & Low Flow). **(D)** Period 4 (High MLH & High Flow). Error bars represent one standard deviation of the mean.

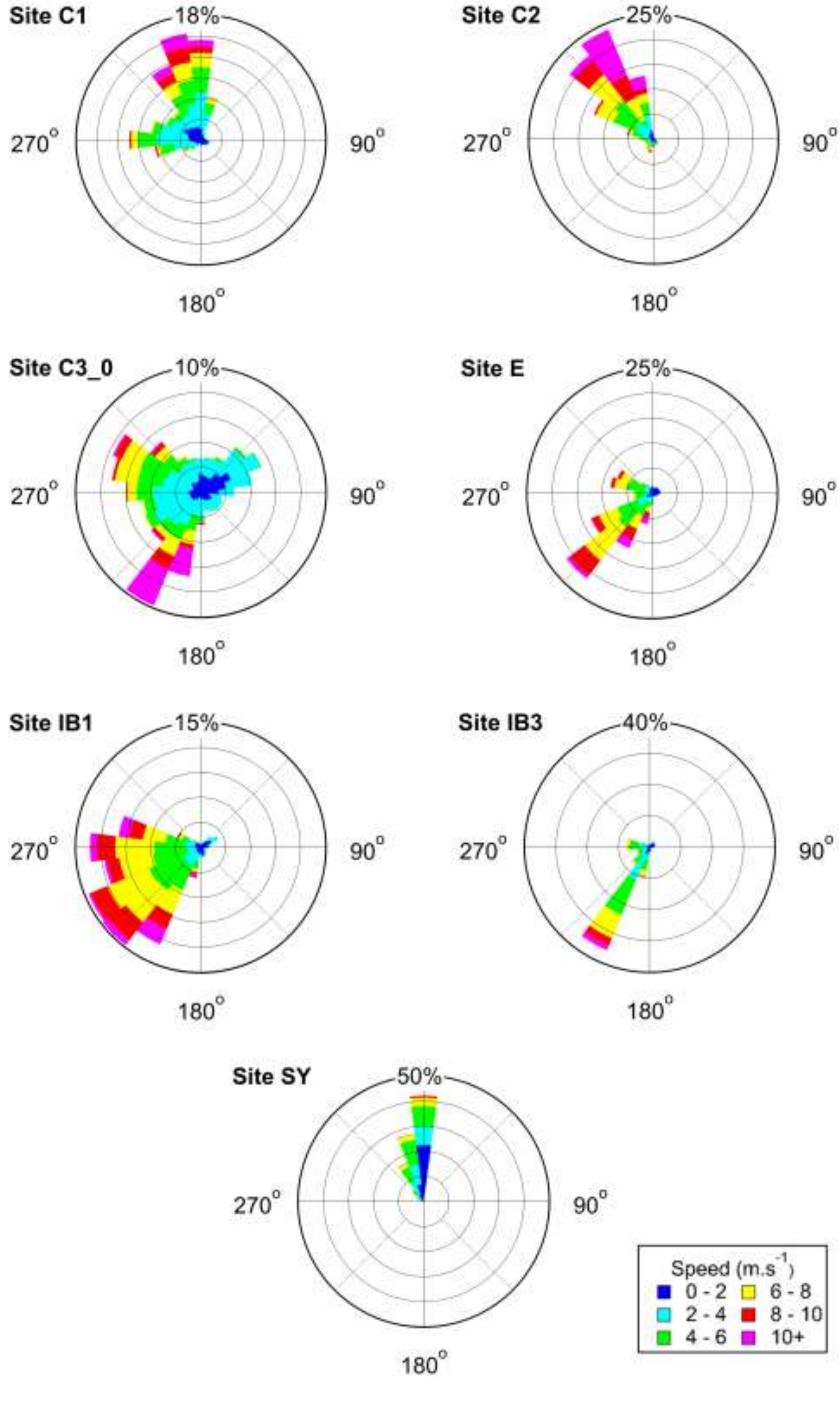

Figure S7. Period 1 (Low MLH & High Flow) daytime localized wind measurements.

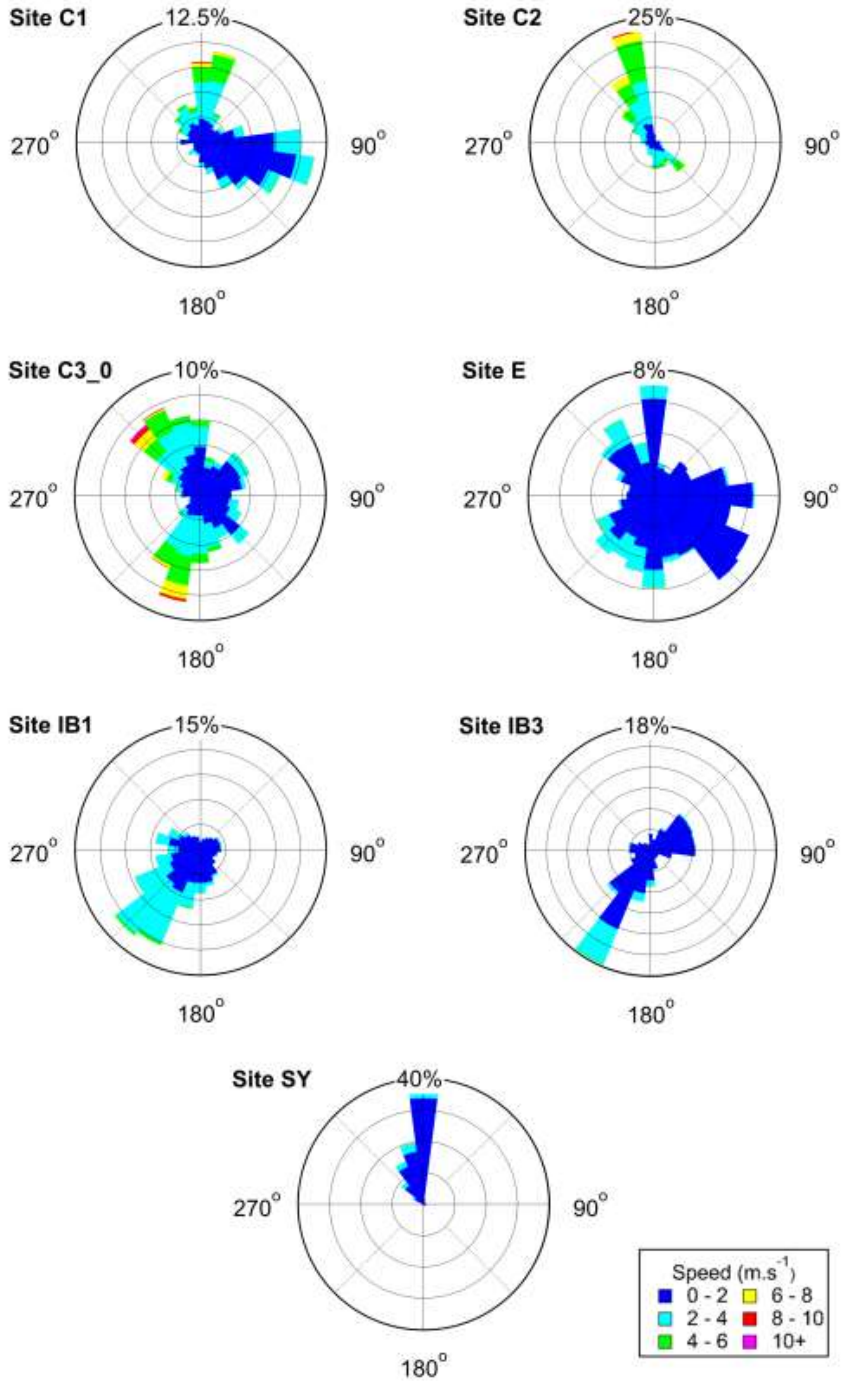

Figure S8. Period 1 (Low MLH & High Flow) nighttime localized wind measurements.

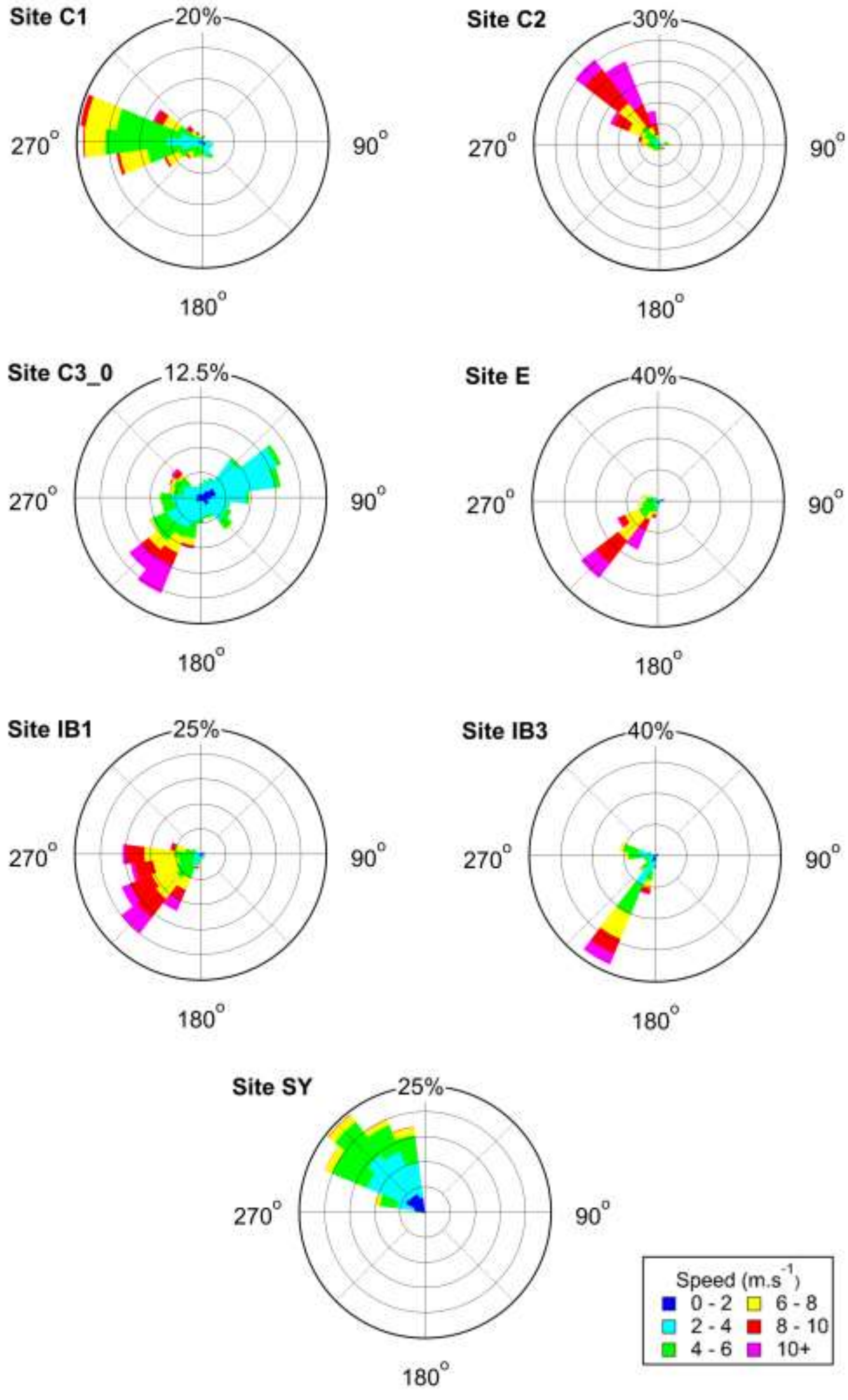

Figure S9. Period 2 (High MLH & Low Flow) daytime localized wind measurements.

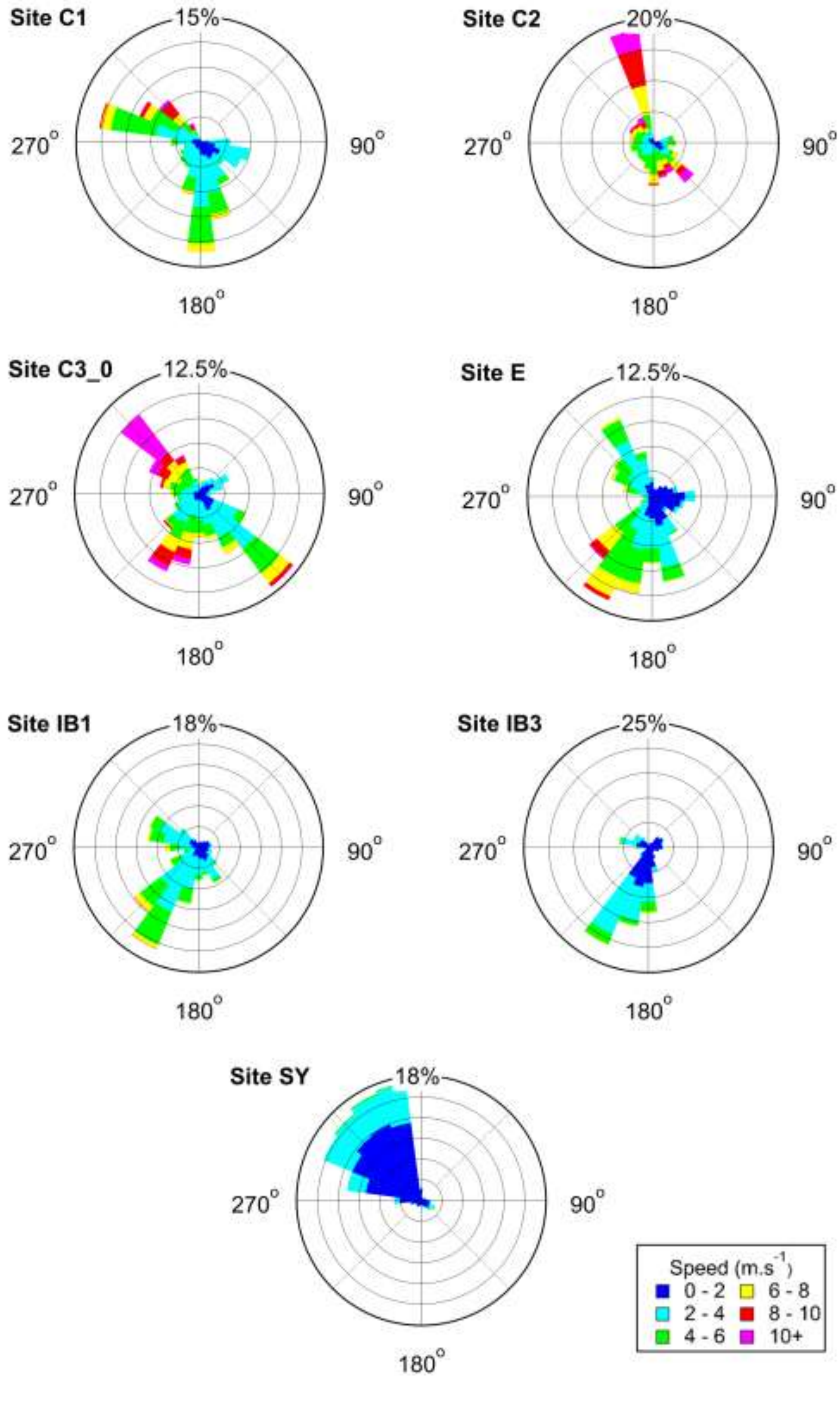

Figure S10. Period 2 (High MLH & Low Flow) nighttime localized wind measurements.

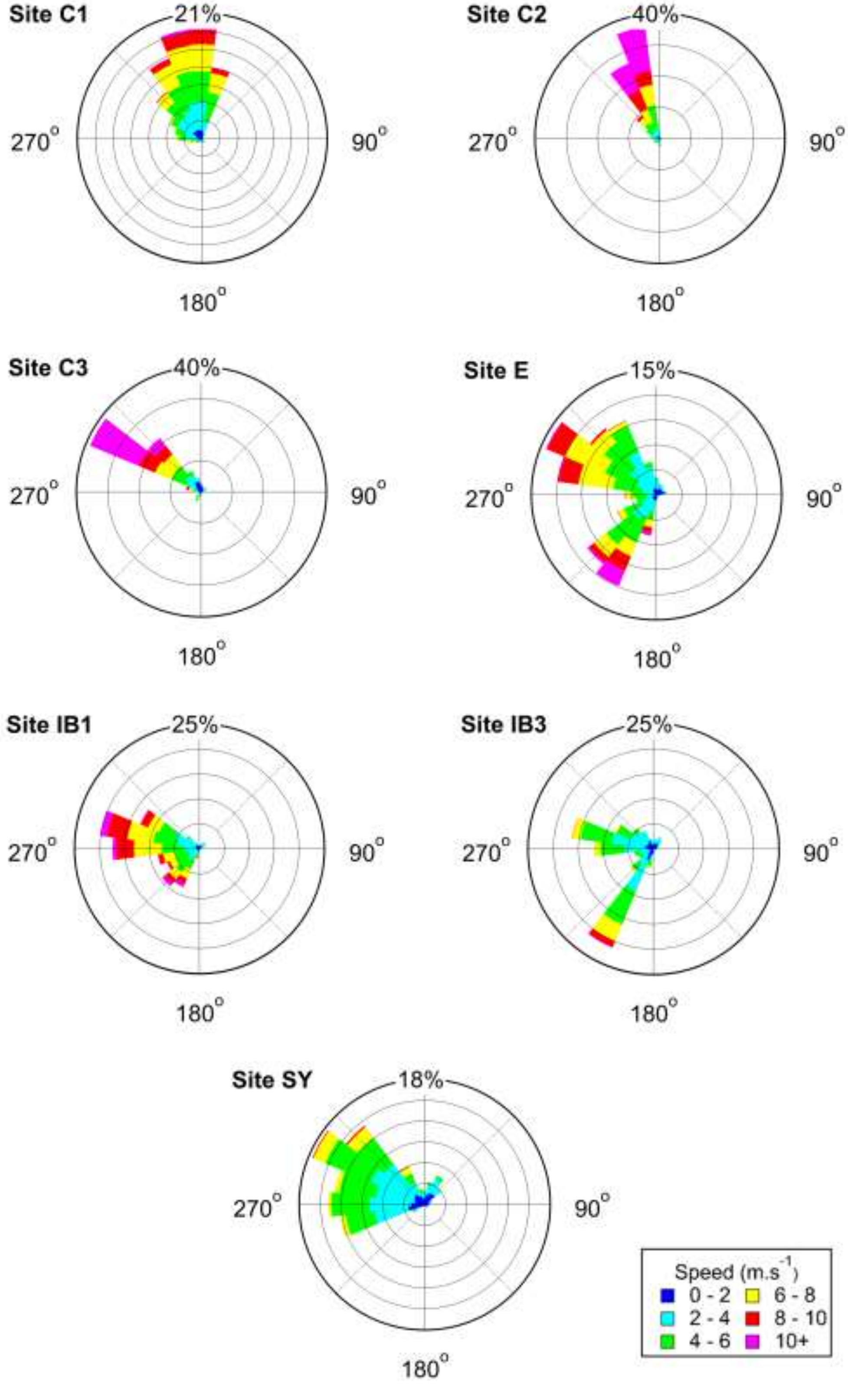

Figure S11. Period 3 (Low MLH & Low Flow) daytime localized wind measurements.

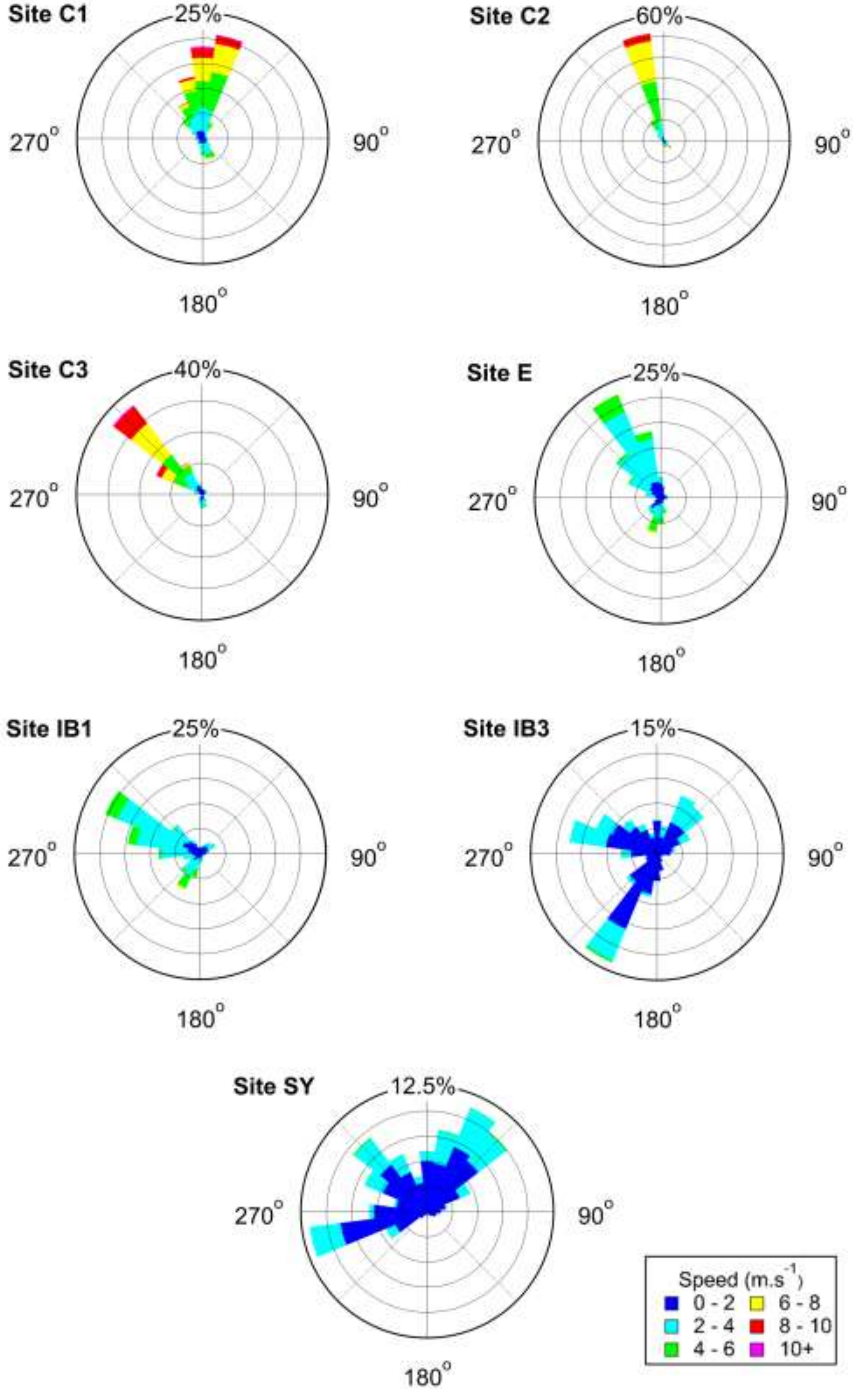

Figure S12. Period 3 (Low MLH & Low Flow) nighttime localized wind measurements.

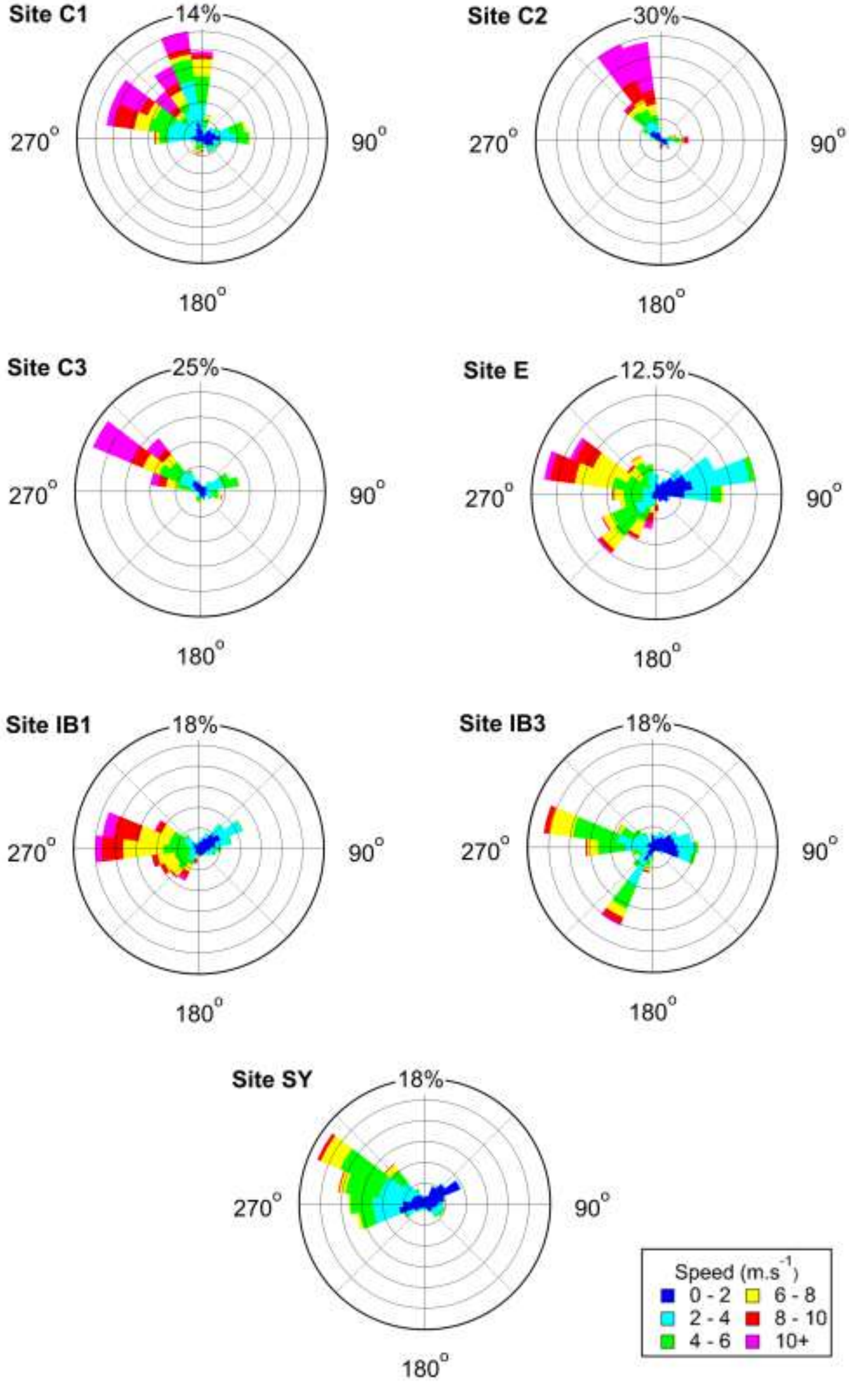

Figure S13. Period 4 (High MLH & High Flow) daytime localized wind measurements.

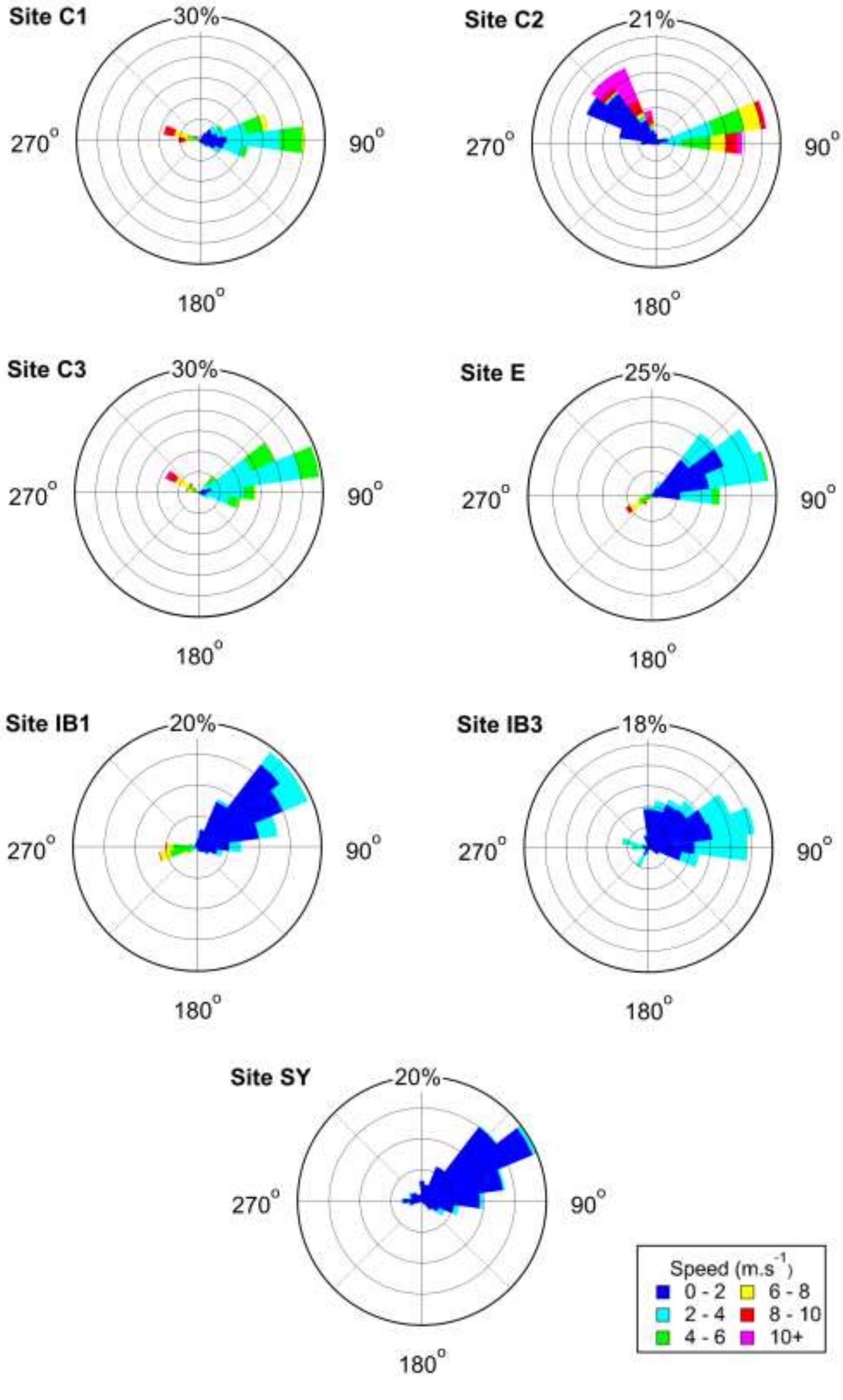

Figure S14. Period 4 (High MLH & High Flow) nighttime localized wind measurements.

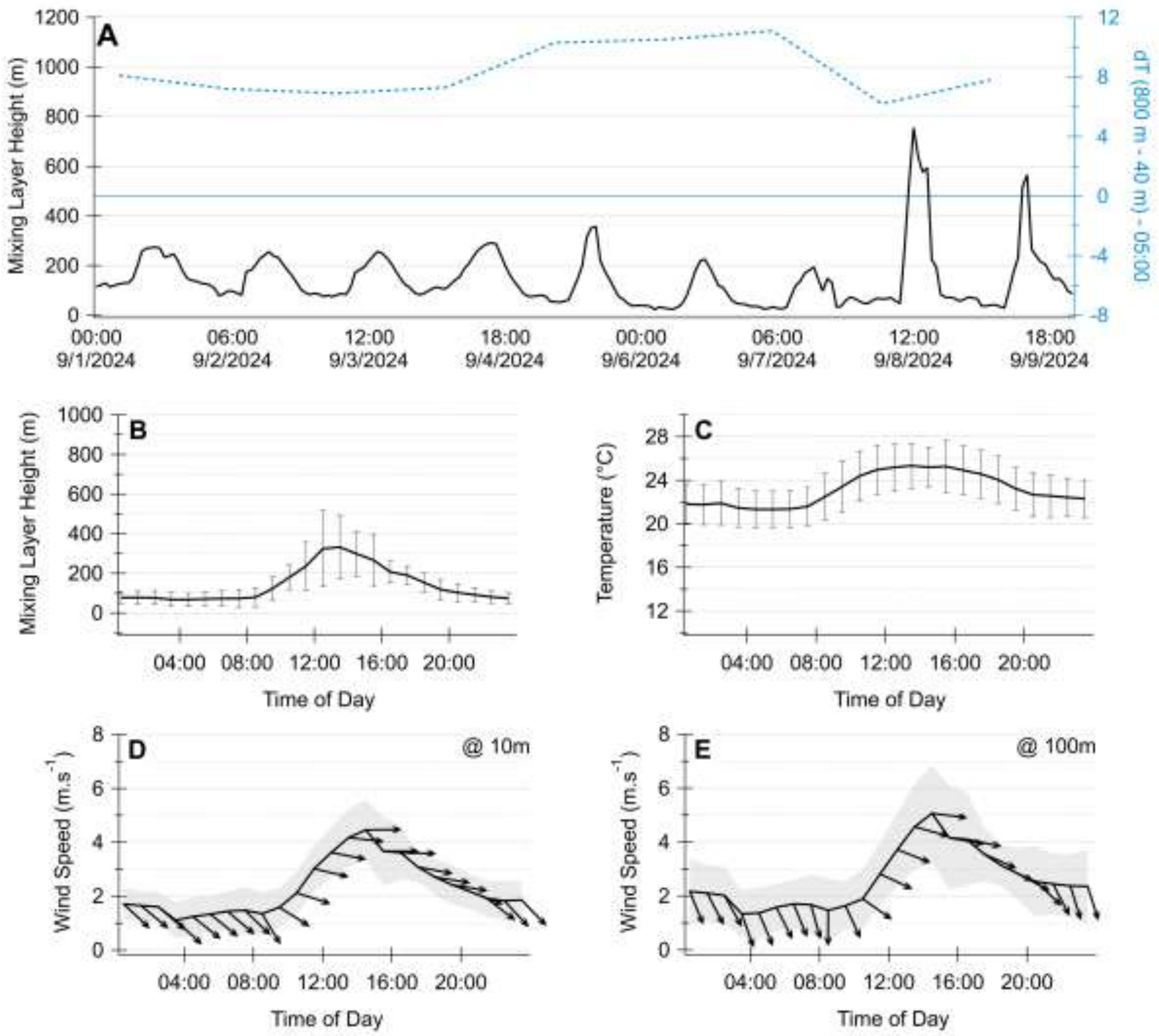

Figure S15. Period 1 (Low MLH & High Flow) ERA5 meteorological parameters. **(A)** Comparison between ERA5 mixing layer height (MLH) and dT estimations. **(B)** MLH diurnal profile. **(C)** Ambient temperature diurnal profile at 2 m. **(D)** Wind diurnal profile at 10 m. **(E)** Wind diurnal profile at 100 m. Error bars and shaded regions represent one standard deviation of the mean.

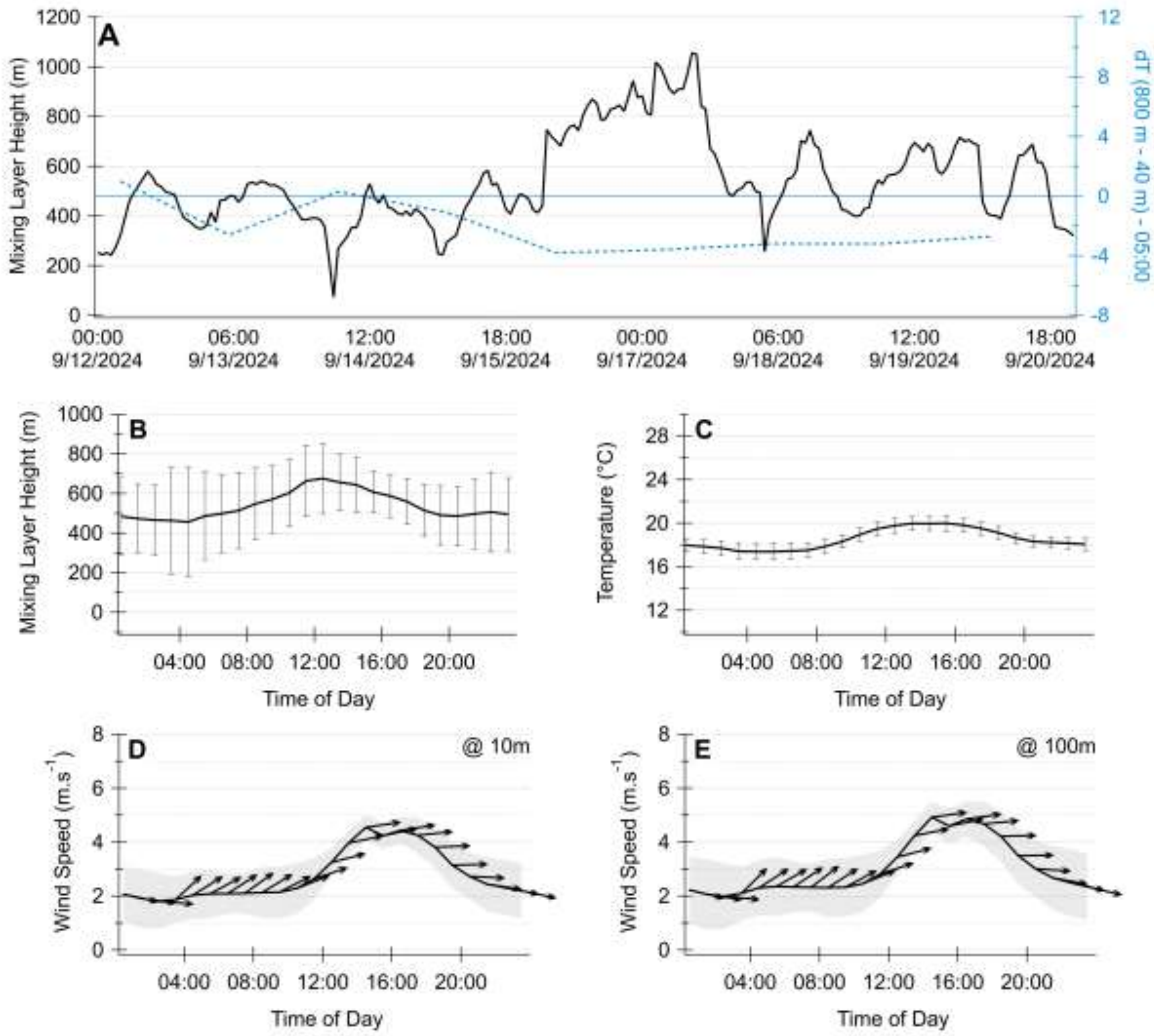

Figure S16. Period 2 (High MLH & Low Flow) ERA5 meteorological parameters. **(A)** Comparison between ERA5 mixing layer height (MLH) and dT estimations. **(B)** MLH diurnal profile. **(C)** Ambient temperature diurnal profile at 2 m. **(D)** Wind diurnal profile at 10 m. **(E)** Wind diurnal profile at 100 m. Error bars and shaded regions represent one standard deviation of the mean.

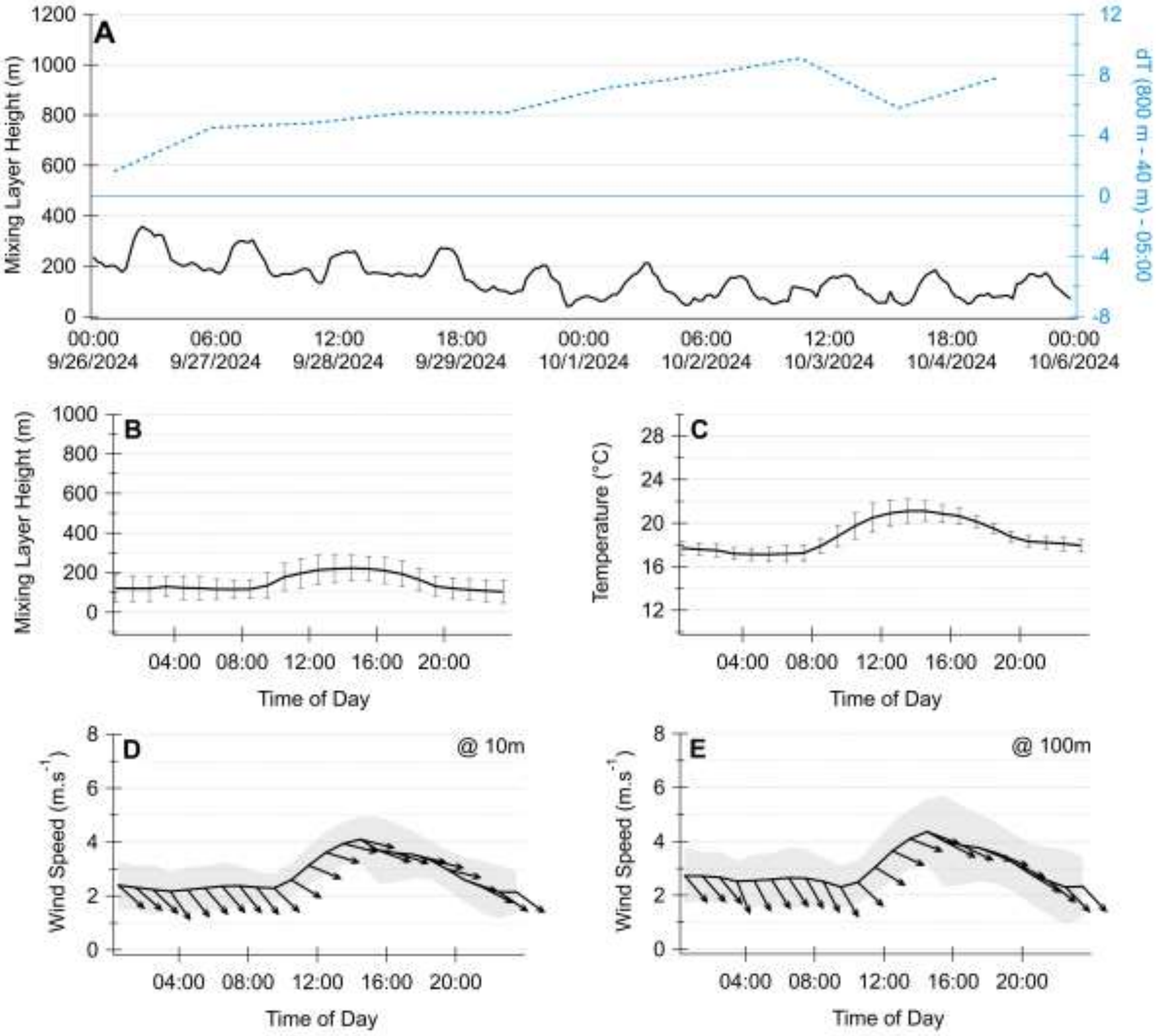

Figure S17. Period 3 (Low MLH & Low Flow) ERA5 meteorological parameters. **(A)** Comparison between ERA5 mixing layer height (MLH) and dT estimations. **(B)** MLH diurnal profile. **(C)** Ambient temperature diurnal profile at 2 m. **(D)** Wind diurnal profile at 10 m. **(E)** Wind diurnal profile at 100 m. Error bars and shaded regions represent one standard deviation of the mean.

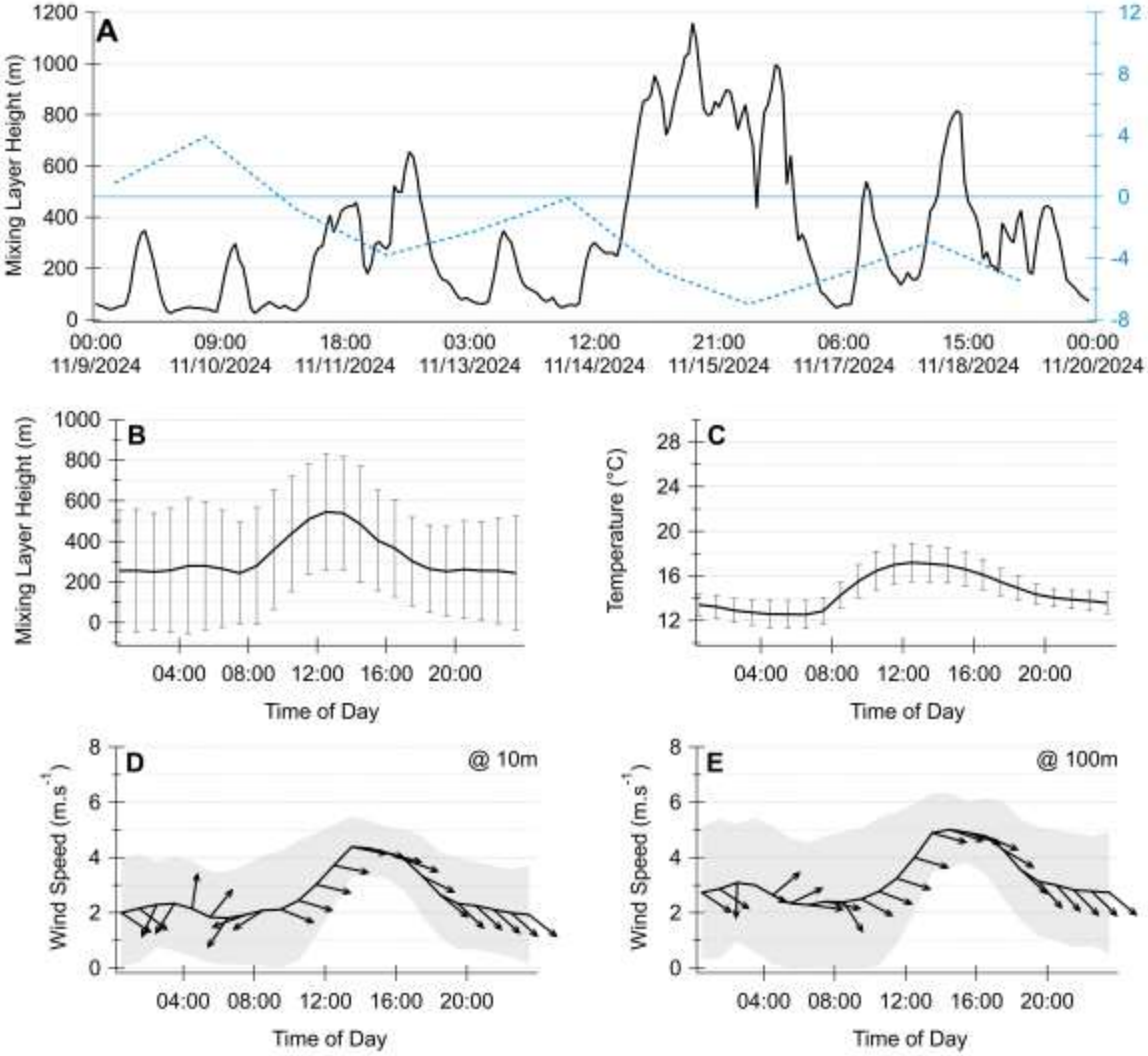

Figure S18. Period 4 (High MLH & High Flow) ERA5 meteorological parameters. **(A)** Comparison between ERA5 mixing layer height (MLH) and dT estimations. **(B)** MLH diurnal profile. **(C)** Ambient temperature diurnal profile at 2 m. **(D)** Wind diurnal profile at 10 m. **(E)** Wind diurnal profile at 100 m. Error bars and shaded regions represent one standard deviation of the mean.

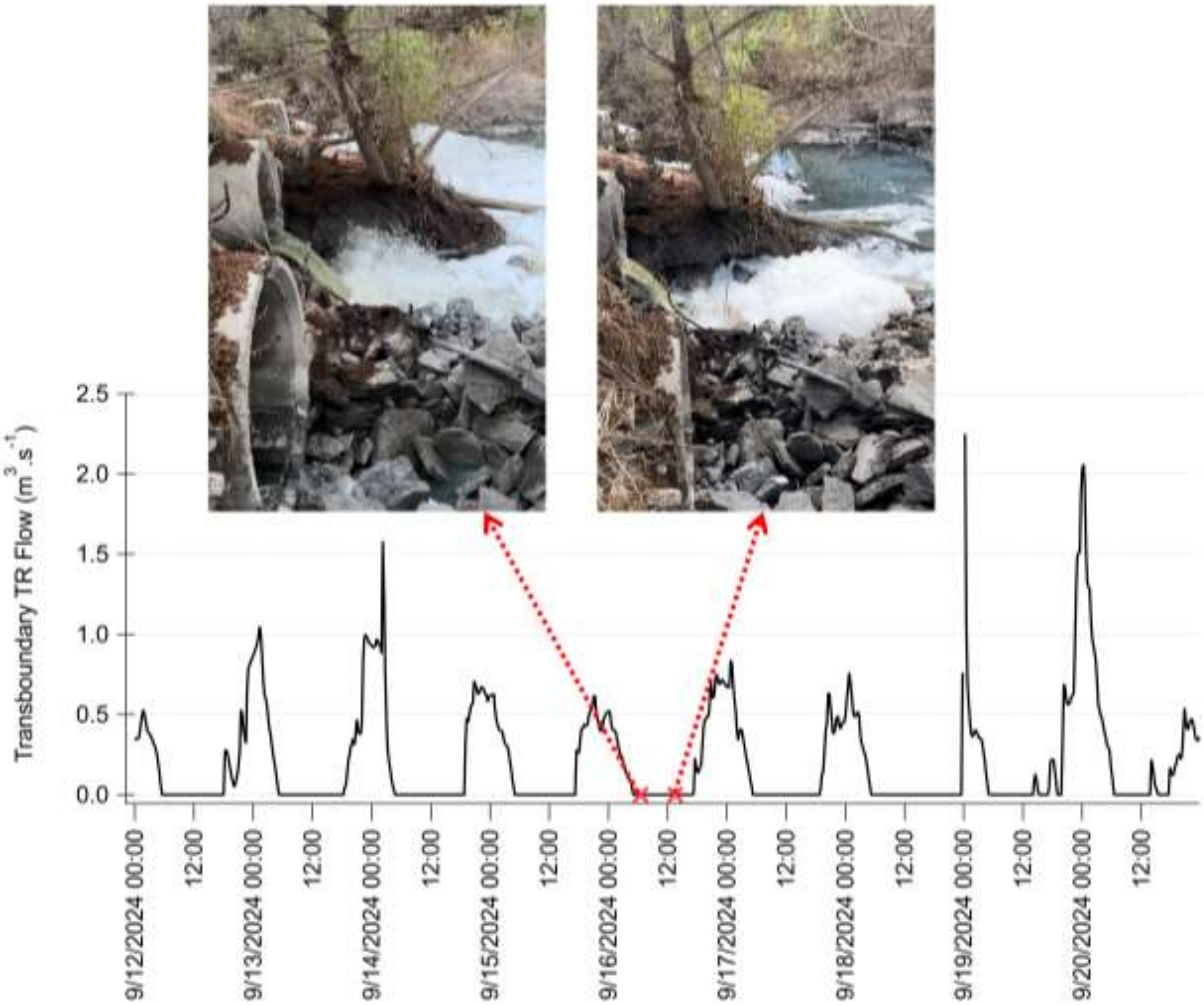

Figure S19. Tijuana River transboundary flow rates measured at the international border. Images show non-zero daytime flows through culvert structures at the turbulent riverine hotspot at Saturn Boulevard (the red "X" in Figure 1) when reported transboundary flows were zero.

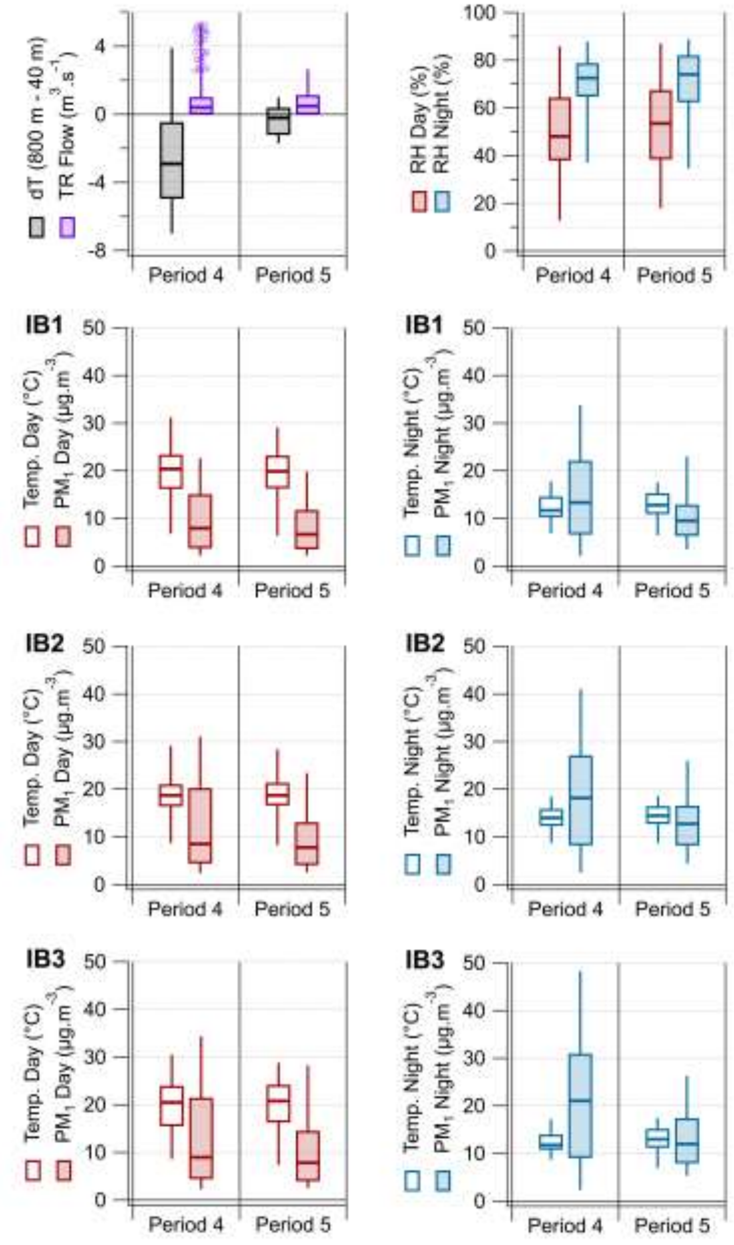

Figure S20. Boxplots comparing PM_1 distributions during Period 4 (Low Temperatures & High Flow) and Period 5 (Low Temperatures & Low Flow) to deconvolute the effect of temperature from the Tijuana River (TR) emissions on PM_1 levels at IB sites. Horizontal lines represent median concentrations. Lower and upper whiskers denote maximum and minimum observations (dT, TR flows, RH, and temperature distributions) and 9th and 91st percentiles (PM_1 distributions). Outliers not included in the PM_1 distributions.

Table S1. Air quality monitoring network details.

Site	Latitude	Longitude	Deployment	Monitoring Device	Data Acquired
C1	32.8663	-117.2546	5/13/2024	MODULAIR™	Temperature, RH, Wind Speed, Wind Direction, PM & Gases
C2	32.6320	-117.1414	8/19/2024	MODULAIR™	Temperature, RH, Wind Speed, Wind Direction, PM & Gases
CV	32.6425	-117.0906	8/27/2024	MODULAIR-PM™	Temperature, RH & PM
C3_0	32.5686	-117.1323	8/23/2024	MODULAIR™	Temperature, RH, Wind Speed, Wind Direction, PM & Gases
C3	32.5806	-117.1325	9/25/2024	MODULAIR™	Temperature, RH, Wind Speed, Wind Direction, PM & Gases
E	32.5748	-117.1267	8/21/2024	MODULAIR™	Temperature, RH, Wind Speed, Wind Direction, PM & Gases
IB1	32.5762	-117.1153	8/14/2024	MODULAIR™	Temperature, RH, Wind Speed, Wind Direction, PM & Gases
IB2	32.5766	-117.0971	8/20/2024	MODULAIR-PM™	Temperature, RH & PM
IB3	32.5654	-117.0905	9/1/2024	MODULAIR™	Temperature, RH, Wind Speed, Wind Direction, PM & Gases
SY	32.5533	-117.0461	8/26/2024	MODULAIR™	Temperature, RH, Wind Speed, Wind Direction, PM & Gases

Table S2. Period 5 meteorological and flow conditions.

Start Period	End Period	Temp. (°C)	RH (%)	dT (m)	TR Flow (m^3 s^{-1})
11/20/24	11/30/24	17.1<br>(7.0 – 28.8)	60.8<br>(17.9 - 88.8)	-0.4<br>(-1.7 – 1.0)	0.7<br>(0 – 2.6)

Table S3. TUV model input.

	**Period 1**	**Period 4**
Latitude	32.5654	32.5654
Longitude	-117.0905	-117.0905
Start Date and Time (LT)	09/01/24 00:00	11/09/24 00:00
End Date and Time (LT)	09/09/24 23:00	11/19/24 23:00
Resolution (hour)	1	1
Measurement Altitude (km)	0.01	0.01
Minimum Wavelength (nm)	280	280
Maximum Wavelength (nm)	420	420
Wavelength Increment (nm)	140	140
Resolution	1 hour	1 hour
Solar Zenith Angle	Calculated by model	Calculated by model
Ozone Column[a] (DU)	289	284
Surface Albedo[b]	0.1	0.1
Cloud Optical Depth[b]	0	0
Cloud Base[b] (km)	4	4
Cloud Top[b] (km)	5	5
Aerosol Optical Depth[b]	0.235	0.235
Single Scattering Albedo[b]	0.990	0.990
Angstrom Exponent[b]	1	1
Sunlight Direct Bean[b]	1	1
Sunlight Diffuse Down[b]	1	1
Sunlight Diffuse Up[b]	NA	NA

[a]Retrived from ERA5 database
[b]Default TUV values

Table S4. Molecular weight and enthalpies of vaporization at 298 K corresponding to each volatility bin considered for the particle phase fraction estimations.

**Saturation Mass Concentration** ($\mu g\ m^{-3}$)	**Molecular Weight**[a] ($g\ mol^{-1}$)	**Enthalpy of Vaporization**[b] ($kJ\ mol^{-1}$)
0.0001	330	123.2
0.001	310	117.4
0.01	290	111.6
0.1	270	105.8
1	250	100
10	225	94.2
100	205	88.4
1000	185	82.6
10000	160	76.8
100000	140	71

[a]Retrieved from Shiraiwa *et al.*[2]
[b]Retrieved from Donahue *et al.*[3]
